# West Nile Virus (WNV): One-Health and Eco-Health Global Risks

**DOI:** 10.3390/vetsci12030288

**Published:** 2025-03-19

**Authors:** Luigi Bruno, Maria Anna Nappo, Raffaele Frontoso, Maria Gabriella Perrotta, Rosanna Di Lecce, Chiara Guarnieri, Luca Ferrari, Attilio Corradi

**Affiliations:** 1 Department of Prevention, Azienda Sanitaria Locale (A.S.L.) Napoli 3 Sud, Castellammare di Stabia, 80053 Naples, Italy; m.nappo@aslnapoli3sud.it; 2 Istituto Zooprofilattico Sperimentale del Mezzogiorno (I.Z.S.M.), Portici, 80055 Naples, Italy; 3 Ministry of Health, Office 3 exDGSAF of the General Directorate of Animal Health, 00144 Rome, Italy; mg.perrotta@sanita.it; 4 Department of Veterinary Science, University of Parma, 43126 Parma, Italy; rosanna.dilecce@unipr.it (R.D.L.); chiara.guarnieri1@unipr.it (C.G.); attilio.corradi@unipr.it (A.C.)

**Keywords:** West Nile virus (WNV), one-health, eco-health, epidemiology, pathogenesis, immune response, clinical signs, pathology, diagnosis, economic impact, surveillance, legislation

## Abstract

West Nile virus (WNV) is an important zoonotic pathogen belonging to the Flaviviridae family, which is endemic in some areas and emerging in others. WNV is transmitted by blood-sucking mosquitoes of the genus Culicoides, Aedes, and Anopheles, and the infection can cause different clinical symptoms. The most common and benign illness in humans is West Nile fever (WNF), but a lethal neurological disease (WNND), related to the neuro-invasiveness of WNV lineage 2, represents the highest health risk of WNV infection. The neuro-clinical form is recognized in mammals (land and cetaceans), particularly in humans (elderly or immunosuppressed) and in horses, avian species, and wildlife animals ranging free or in a zoological setting. This review highlights the most relevant data regarding epidemiology, virology, pathogenesis and immunity, clinical signs and differential diagnosis, pathology and imaging, histopathology and gross pathology, economic impact, influence of climate change, and surveillance of WNV. Climate change has favored the wide spread of WNV in many areas of the globe and consequent One-Health and Eco-Health emergencies, influencing the health of human beings, animals, and ecosystems.

## Simple Summary

West Nile virus (WNV) causes mild fever or, in the most severe cases, encephalitis and meningitis in humans and animals due to its ability to cross the blood–brain barrier. The virus is endemic or epidemic in every geographical area of the world, except for Antarctica. Birds, especially wild birds, serve as the definitive host, and it is transmitted to humans, horses, and wild animals through blood-sucking mosquito species. Climate change has facilitated the global spread of WNV, resulting in One-Health and Eco-Health emergencies, as well as in an economic impact.

## 1. Introduction

Flaviviruses comprise a group of 90 viruses responsible for causing diseases in both humans and animals [1]. The Flaviviridae family includes the genera Orthoflavivirus, Hepacivirus, Pestivirus, and Pegivirus [2]. These genera encompass numerous pathogens significant to both human and animal health, such as West Nile virus (WNV), Yellow fever virus (YFV), and Dengue virus (DENV) [3]. The genus Orthoflavivirus includes 53 species that can be categorized into tick-borne and mosquito-borne species, both of which affect human and animal health [4].

West Nile virus (WNV) belongs to the Orthoflavivirus genus, Flaviviridae family, and is an ubiquitous infectious agent responsible for worldwide outbreaks transmitted by mosquitoes [5]. It is a neurotropic virus that infects both the central and peripheral nervous systems [6] and is a member of the Japanese Encephalitis complex [7].

WNV is believed to have originated in Africa, with its first recorded identification in Uganda in 1937 [8]. WNV has caused numerous outbreaks in both human and animal populations across Africa, Europe, Asia, and the Americas [9,10,11,12]. Phylogenetic analyses suggested that it emerged between the 16^th^ and 17^th^ centuries [8,13,14]. Subsequent outbreaks were reported in Israel and Egypt in 1951 (lineage 1) [15,16]. WNV was later detected in Europe, with cases reported in Albania in 1958 and in France in 1962 [17,18]. A significant outbreak occurred in Romania in 1996, with an estimated mortality rate of 4% [19,20]. Subsequent outbreaks included one in Russia in 1999, with 480 human cases and a 10% mortality rate [21], and the first detection of WNV in the United States of America (USA), in New York City (NYC) in 1999 [22,23]. Additional outbreaks occurred in Europe in the early 2000s, associated with lineage 1, which circulated in the southern regions of the continent. A notable shift occurred in 2004, with the detection of lineage 2 in a goshawk (*Accipiter gentilis*) in Hungary [24]. This lineage rapidly spread to other southern European countries, with significant outbreaks involving large numbers of human cases, particularly in Greece in 2010 [25].

The spread of the disease in Europe has also been associated with climate changes recorded in recent years, which facilitated the introduction of the West Nile disease (WND) lineage 1 [26]. In the continent, WNV continues to pose a public health threat annually, with frequent outbreaks. The largest outbreak, reported in 2018, was driven by the concurrent circulation of lineages 1 and 2, which recently extended beyond endemic boundaries into northern regions [27]. There is evidence of emerging or re-emerging WNV in high-latitude regions and at the edges of current endemic zones [28,29,30,31,32]. In Europe, the increase in WNV cases and the emergence of new outbreak locations are expected in relation to new climate scenarios, especially on the boundaries of the current transmission areas [30]. In Germany, the extreme summer temperatures of 2018 likely played a significant role by shortening the average incubation period in mosquitoes, facilitating rapid viral amplification and increasing the risk of transmission to vertebrate hosts [31]. Nevertheless, the impact of climate change on WNV distribution can vary geographically, with some areas potentially experiencing a reduction in cases [33].

The primary route of WNV transmission to humans is through the bite of infected mosquitoes during blood meals. WNV can infect a wide range of vertebrate species, including many mammals, birds, and some reptiles and amphibians [34,35]. However, the virus replicates and amplifies its transmission cycle primarily in mosquitoes and birds, with migratory birds playing a crucial role in its spread [36]. In Italy, species such as the blackbird (*Turdus merula*), magpie (*Pica pica*), thrush (*Turdus* spp.), and house sparrow (*Passer domesticus*) are the main bird species implicated in WNV spread [37]. In the USA, the American robin (*Turdus migratorius*) is considered one of the primary host species [38]. Humans and horses become infected in an incidental manner through mosquito bites and are considered dead-end hosts due to insufficient viremia to sustain further transmission [39,40,41]. Outbreaks in wild birds and horses often precede human infection reports and are considered useful indicators of an impending human WND epidemic season [24,42]. The distribution of vectors necessary for transmission depends on environmental conditions and is influenced by climate changes [43,44]. In experimental animals, aerosol transmission has also been reported [45].

WNV is maintained within a complex enzootic cycle between mosquitoes and birds, with more than 300 avian species involved as vectors and hosts [46]. CNS involvement occurs in certain individuals through hematogenous or trans-neuronal routes (retrograde axonal transport via the olfactory nerve or peripheral nerves) [12,47]. In both humans and horses, 80% of infected individuals are asymptomatic [48]. There are three potential manifestations of WND infection in humans, influenced by host immunity and viral strain: approximately 80% of human infections are asymptomatic, while West Nile fever (WNF) presents flu-like symptoms. A notable feature of some Orthoflaviviruses, such as WNV, is neuro-invasiveness [49,50]. The least common manifestation of WND is the neuroinvasive form (West Nile neuroinvasive disease, WNND), which affects approximately 0.04% to 0.07% of infected individuals. Of particular interest are the psychiatric and cognitive aspects of the disease [51].

Most horses with clinical manifestations exhibit fever and lethargy [48,52]. A smaller proportion develops neurological signs due to meningoencephalitis, involving inflammation of the brain and meninges. Neurological signs include ataxia, blindness, paralysis, and muscle fasciculations. In humans presenting neurological signs, mortality rates reach up to 20%, whereas in horses, the rates are as high as 30%. An additional 10–20% of horses exhibit persistent neurological deficits following recovery [48]. One of the risk factors for developing severe disease is age. Patients over 65 years old are 16 times more likely to develop severe disease than younger individuals, while those over 70 years old have a 30- to 45-fold-increased risk compared to younger patients [53,54]. Other risk factors include male sex and various immunocompromised conditions [55,56]. WNV infection is often associated with occupational exposure, particularly for workers in environments with birds or other animals and those working outdoors [57]. Changes in outdoor work organization due to rising temperatures, such as increased work at dawn and dusk, increase the risk of mosquito bites, as these times coincide with peak mosquito activity [58]. Veterinarians are a potential at-risk group: in a study where 60% of horses tested positive for the disease, 23% of veterinarians also had serological evidence confirming WNV infection [59].

WND represents an excellent case study for the One-Health approach. Orthoflaviviruses are distributed globally across all continents except Antarctica [50,60]. The number of WNV infection cases has risen not only in the USA [61] but also in Europe, particularly in Central and Mediterranean regions [24,62]. In 2023, 2566 cases were reported in the USA [62], and approximately 700 cases were observed in Europe, with Italy reporting the highest frequency (366 cases) [63]. In recent years, WND infection cases have increased in number and spread to new territories. The primary driver of this variable epidemiology is climate change, which influences global climatic parameters, such as temperature and humidity. These changes enhance environments suitable for mosquito proliferation and alter migratory routes of birds [12,64,65]. Over the past two decades, climate change has likely impacted ecosystems, leading to shifts in the natural habitats of many animal species [44,66]. Rising temperatures correlate with increased insect metabolism and shortened life cycles. Consequently, mosquito activity intensifies, resulting in more frequent blood meals and higher transmission rates of various pathogens, including those in the Orthoflavivirus genus that cause disease in humans and numerous wild and domestic animals [67]. Increasingly, systematic reviews confirm that environmental factors and climate changes significantly influence virus distribution, transmission dynamics, and mosquito habitats [26,68]. High temperatures accelerate viral replication, shorten the incubation period in vectors, promote mosquito abundance, enhance transmission efficiency, and increase the availability of favorable mosquito habitats. They also increase the likelihood of avian migration between regions [69,70]. Moreover, altered precipitation patterns significantly impact mosquito reproduction and abundance, affecting WNV distribution [43]. Strong evidence now exists that climate change directly influences the proliferation of vector-borne diseases, including WND [44,71]. Numerous studies demonstrate that areas susceptible to WNV transmission may expand or shift due to climate change. This includes projections of future global climate scenarios, laboratory studies on how carrier species respond to environmental changes, and field research in regions experiencing epidemics [33].

Currently, no vaccines or specific treatments are available for humans [72,73,74]. Therefore, preventive measures focus on vector control campaigns and general guidelines for protection against mosquito bites [75,76,77].

In a world where human, animal, and environmental health are increasingly interconnected, WND emerges as an invisible yet tangible threat. This is not solely a human issue: the virus crosses subtle boundaries between species and habitats, underlining the intricate interdependence of our ecosystem health (Eco-Health). While society embraces the comfort of modern cities, threats like this continue to thrive in the seams of our shared environment. What are the intersections between nature, urbanization, and health risks? And how can we tackle a challenge that demands coordinated solutions across diverse disciplines? The authors explore why WND represents one of the most emblematic challenges of the One-Health paradigm in addition to Eco-Health, as well as the actions to be taken to counteract the local and global spread of WNV mainly through its surveillance and monitoring and immunoprophylaxis in horses.

The aim of this review is to present the most relevant data regarding epidemiology, virology, pathogenesis and immunity, clinical signs and differential diagnosis, pathology and imaging, histopathology and gross pathology, economic impact, influence of climate change on Eco-Health, and surveillance of WNV, as well as updates on the European regulations in both the medical and veterinary fields.

## 2. Epidemiology

WNV ranks among the most prevalent arboviruses, presenting major public and veterinary health risks and challenges. WNV has spread throughout the world, except for Antarctica, and is now considered the most important causative agent of human viral encephalitis worldwide [9]. Research indicates that WNV is endemic in Europe, where it has circulated for decades through natural cycles involving mosquitoes as vectors and various hosts such as birds, horses, and humans. Among the primary vectors, mosquitoes of the *Culex* (*Cx.*) *pipiens* complex are particularly important due to their widespread distribution, even in densely populated urban areas, which complicates effective control measures [76,78]. Antibodies against WNV have been detected in various hosts, including humans and horses, in multiple regions. At the same time, the virus has also been isolated from both vector species and deceased host animals. The epidemiological landscape in Europe and influences such as climate change and differing precipitation patterns could encourage the growth of competent mosquito species or the introduction of invasive ones. This may heighten the risk of WNV outbreaks and the transmission of other vector-borne diseases. WNV transmission occurs primarily through mosquito bites, with mosquitoes acquiring the virus from viremic birds. The transmission intensity to humans and equines is influenced by mosquito populations’ density, infected birds’ prevalence, viral load, and specific ecological and environmental conditions. These variables explain the diverse epidemiological patterns observed over time. For instance, specific African outbreaks reported symptomatic cases in up to 55% of the population. In contrast, more recent epidemics in Europe, the Middle East, and North America have shown lower attack rates, typically affecting 3% to 5% of populations in areas with viral circulation [79]. A notable characteristic of WNV is its exceptional ability to adapt to different environmental conditions, with infections reported in a range of ecosystems. The virus’ broad host diversity, including mammals, birds, and reptiles, enhances its widespread presence and survival in various ecosystems.

WNV transmission has been observed in Europe, the Middle East, Africa, India, Australia (where the Kunjin virus [WNV_KUN_], a WNV subtype, was found), North America, and some areas of Asia, Central America, and the Caribbean. Recent cases of WNF in humans were reported predominantly in Mediterranean countries, including Algeria, Morocco, Tunisia, Romania, and the Czech Republic, as well as in Israel, Russia, North America, and France [62,63,80,81,82]. Outbreaks affecting equines have been reported in Morocco, Italy (1998–2024), North and South America (since 1999), Israel, and France [9,79,83]. In temperate zones, WNV spread is seasonal, peaking between July and October, although some US regions report cases from mid-April to December. Birds, particularly wild species, are the primary vertebrate hosts. WNV has been isolated from over 150 species of domestic and wild birds worldwide [84].

WNV has been classified into nine lineages based on biology, evolution, pathogenicity, and geographic distribution [85,86,87]. The most virulent strains are found in lineages 1 and 2, associated with numerous outbreaks of severe neurological disease worldwide [24]. Lineage 1 is further subdivided into three sub-lineages: sub-lineage 1a, which includes isolates from Africa, Europe, and the Middle East; sub-lineage 1b, consisting of WNV_KUN_ strains from Australasia; sub-lineage 1c, also referred to as lineage 5, which encompasses isolates from India. Lineage 2, although neurotropic, exhibits lower virulence compared to lineage 1. This lineage includes isolates from Sub-Saharan Africa, Madagascar, and Europe and has been implicated in various outbreaks affecting humans, horses, and birds [12,88].

## 3. Viral Life Cycle and Transmission

WNV is maintained in nature through a primary mosquito–bird–mosquito transmission cycle. The WNV life cycle involves three key groups: virus reservoirs, primarily birds that harbor the virus without exhibiting clinical signs; mosquito vectors, which support viral replication; and final or incidental hosts, such as humans, horses, and other mammals, which become infected during a mosquito blood meal. They are unable to amplify the disease or serve as reservoirs. These hosts, often called “dead-end hosts”, do not play a significant role in virus amplification. However, crocodilians are an exception, as they can amplify the virus [89].

The transmission cycle involves the following steps:

1. Infection of the mosquito after feeding on a viremic bird;
2. Viral replication and dissemination within the mosquito body;
3. Transmission of the virus from the mosquito to a susceptible vertebrate through another blood meal [90].

Competent mosquito vectors acquire WNV by feeding on a viremic vertebrate host. Once ingested, the virus reaches the mosquito’s midgut, replicating it and spreading to the salivary glands. This enables the mosquito to transmit the virus to another host during its next blood meal. Interestingly, as with other Orthoflaviviruses, WNV does not cause apparent disease in mosquitoes [91]. After replication in the midgut and other tissues, the virus moves retrogradely to the salivary glands through the hemolymph.

The accumulation of the virus in the salivary glands eventually leads to high levels of viremia in the saliva, from which it can subsequently be transmitted to another host during feeding [38].

Infection occurs when the mosquito saliva contains a viral load exceeding 10^4^ TCID_50_/mL, potentially leading to clinical disease in the host [38]. Vertebrate hosts —both reservoirs and incidental hosts—become infected when bitten by a WNV-infected mosquito. While probing for a suitable feeding site, mosquitoes inject saliva into the host’s blood vessels before drawing a blood meal [12].

The cycle can further be divided into rural/wild (more common) and synanthropic/urban cycles. The virus is transmitted between migratory wild aquatic birds and ornithophilic mosquitoes near river deltas and marshy wetlands. The high viremia levels and persistence of WNV in some bird species (up to 100 days) explain its spread from endemic to non-endemic areas during migrations. In urban cycles, domestic/synanthropic birds and mosquitoes that can feed on birds and mammals, including humans, are involved; examples include *Cx. pipiens* and *Cx. modestus*. Notable urban transmission cases occurred in Bucharest (Romania, 1996–1997) and NYC (USA, 1999). Direct transmission has been observed in animals fed infected carcasses, as with alligators in the USA (2001–2002) [89], and experimentally in some raptor species. In birds, transmission through contact with excretions and secretions has been hypothesized, particularly during mating season. Vertical transmission has been demonstrated in mosquitoes (e.g., Culex, Ochlerotatus, Aedes, Coquillettidia, Anopheles) [92].

Humans can also acquire WNV through (1) transfusion of infected blood, (2) organ transplants from infected donors, (3) breast milk, and (4) vertical transmission from mother to fetus [20,83,93]. In experimental animals, aerosol transmission has also been reported [45].

Primary vectors are ornithophilic mosquitoes of the Culex genus. In Europe, *Cx. pipiens* and *Cx. modestus* are primary vectors, but in Russian hot and dry southern regions, WNV has been isolated in ticks (i.e., Ixodes, Hyalomma marginatum, Ambylomma, Dermacentor) [94,95,96]. WNV has been isolated in 62 mosquito species across 10 genera in the USA.

Serological surveys have confirmed the circulation of WNV in Europe since the 1950s [17].

The first documented outbreak in humans occurred in the Camargue region of southern France during 1962–1963. The epidemic in Romania 1996 marked the first significant outbreak of WNV in Europe, with approximately 400 cases reported [19,97]. Since then, cases and outbreaks have been recorded in southern, eastern, and western European countries. WNV transmission in Europe is influenced by factors such as the level and duration of viremia in the host, vector and host species, the number of mosquitoes capable of becoming infected, climatic conditions, and the presence and concentration of susceptible hosts.

Temperature is considered a critical environmental factor influencing WNV activity in Europe. Higher-than-normal summer temperatures facilitate the spread and amplification of the virus into new areas by affecting mosquito breeding and the extrinsic incubation period of the virus [97]. A study highlighted that July temperature anomalies (high temperature), adequate water surfaces in June, the presence of wetlands, proximity to migratory bird routes, and prior WNV circulation are strongly associated with new cases in Europe [98].

Climatic factors are the primary determinants of the population dynamics of mosquitoes that transmit WNV, with temperature and prolonged periods of moderate-to-warm weather being the most significant drivers of mosquito population growth. Warmer climate in Europe generally results in a shorter incubation period for WNV and accelerates the virus evolutionary rate, consequently increasing the viral load within host populations. Furthermore, at higher temperatures, Culex mosquitoes develop more quickly, extend their reproductive season, and feed more frequently. Therefore, rising temperatures likely lead to faster transmission and a broader distribution of WNV, longer transmission seasons, and an elevated risk of local human WNV infection in both transmission areas and previously unaffected European regions [99]. Before 2004, sporadic cases and occasional outbreaks in animals and humans in Europe were primarily linked to lineage 1 WNV strains. However, a lineage 2 strain emerged in Hungary in 2004 and began spreading across central Europe and the eastern Mediterranean region in 2008. This lineage caused significant outbreaks in Greece, Hungary, and Serbia. Another lineage 2 strain appeared in southern Russia in 2007 and later spread to Romania and Italy from 2010 onwards [25,88]. In certain countries, including Italy, Romania, and Turkey, neurovirulent WNV strains from multiple genetic lineages are known to circulate simultaneously.

## 4. Geographical Distribution

**Figure 1:**
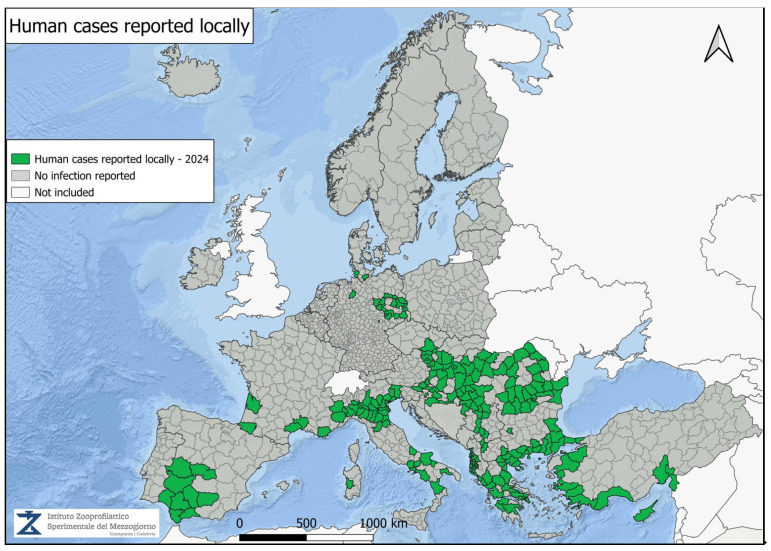
Distribution of locally acquired human WNV infections in 2024 in the European Economic Area (EEA). The EEA is divided into smaller areas according to the classification known as the Nomenclature of Terrestrial Units for Statistics (NUTS), and specifically NUTS3 refers to the Regional Level, sourced from EUROSTAT 2024 (https://ec.europa.eu/eurostat/web/nuts; accessed on 1 March 2025).

Figure 1 presents the final seasonal update for WNV in 2024 [62]. A total of 19 countries in Europe have reported locally acquired human cases of WNV infection since the beginning of the year: Albania, Austria, Bulgaria, Croatia, Cyprus, Czech Republic, France, Germany, Greece, Hungary, Italy, Kosovo, North Macedonia, Romania, Serbia, Slovakia, Slovenia, Spain, and Turkey. WNV infection in humans is classified as a notifiable disease at the European level. According to the European case definition, National Public Health Authorities are required to report cases through The European Surveillance System (TESSy). Outbreaks of WNV infections in wildlife and domestic animals must be reported to the Animal Disease Information System (ADIS) of the European Commission (EC). At the EU/EEA (European Union/European Economic Area) level, it is mandatory to report equine encephalomyelitis caused by WNV infection and WNV infections in birds, in accordance with Commission Implementing Regulation (EU) 2018/1882. All these data are submitted by the relevant Veterinary Services through the World Animal Health Information System (WAHIS) to the World Organization for Animal Health (WOAH) [100].

**Figure 2:**
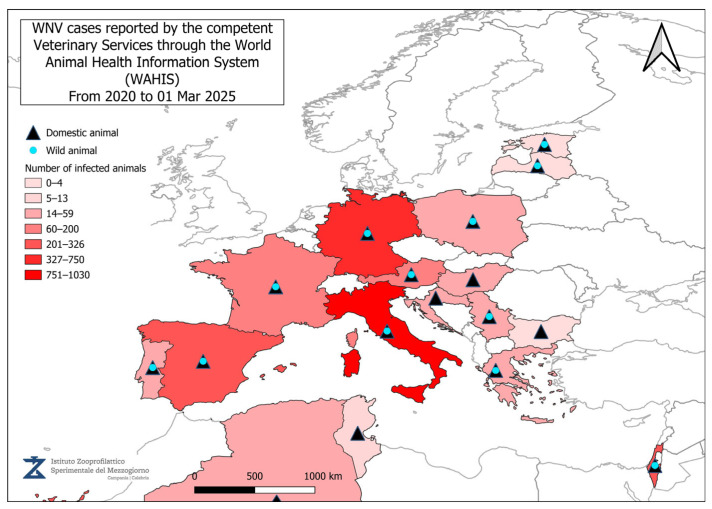
WNV cases submitted by the relevant Veterinary Services through WAHIS in Europe. Distribution of the WNV cases in Europe, reported by the relevant Veterinary Services through WAHIS from 1 January 2020 to 1 March 2025. The number of infected animals is depicted using a color scale ranging from light pink (0–4 cases) to bright red (751–1030 cases). Symbols indicate the types of animals: light blue circles indicate wild animals, while black triangles indicate domestic animals.

**Figure 3:**
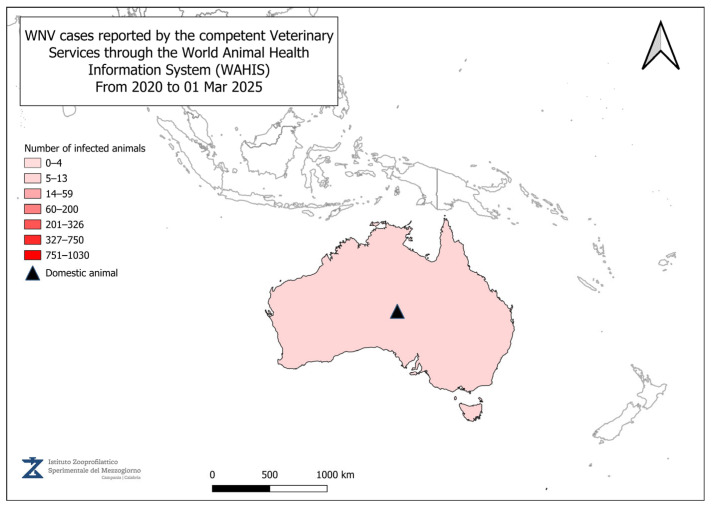
WNV cases submitted by the relevant Veterinary Services through WAHIS in Oceania. Distribution of WNV cases in Oceania, reported by the relevant Veterinary Services through the WAHIS from 1 January 2020 to 1 March 2025. The number of infected animals is depicted using a color scale ranging from light pink (0–4 cases) to bright red (751–1030 cases). Symbols indicate the types of animals: black triangle indicates domestic animals.

**Figure 4:**
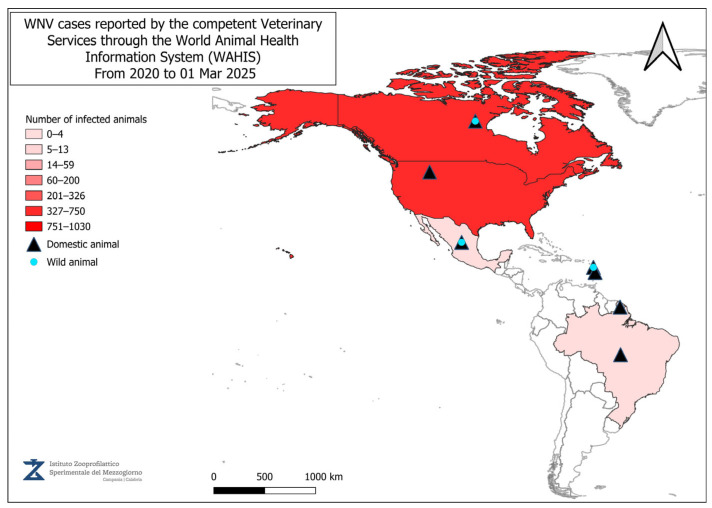
WNV cases submitted by the relevant Veterinary Services through WAHIS in Americas. Distribution of WNV cases in the Americas, reported by the relevant Veterinary Services through WAHIS from 1 January 2020 to 1 March 2025. The number of infected animals is depicted using a color scale ranging from light pink (0–4 cases) to bright red (751–1030 cases). Symbols indicate the types of animals: light blue circles indicate wild animals, while black triangles indicate domestic animals.

**Figure 5:**
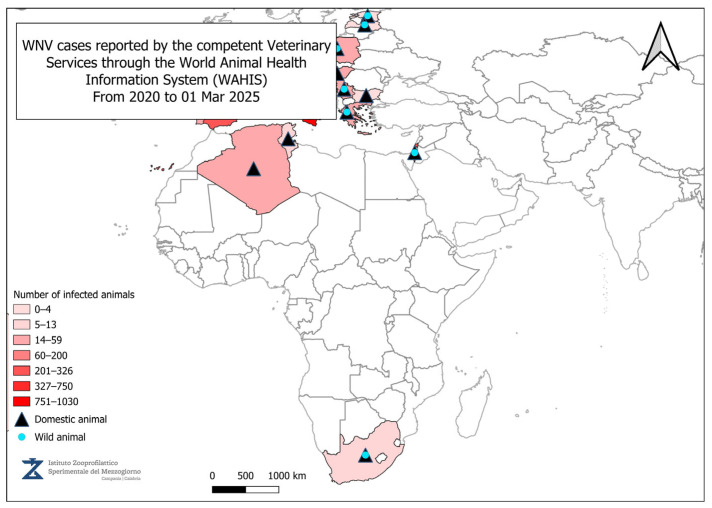
WNV cases submitted by the relevant Veterinary Services through WAHIS in Africa and Asia. Distribution of WNV cases in Africa and Asia, reported by the relevant Veterinary Services through WAHIS from 1 January 2020 to 1 March 2025. The number of infected animals is depicted using a color scale ranging from light pink (0–4 cases) to bright red (751–1030 cases). Symbols indicate the types of animals: light blue circles indicate wild animals, while black triangles indicate domestic animals.

Figure 2, Figure 3, Figure 4 and Figure 5 present data from the official reports submitted by the relevant Veterinary Services through WAHIS, in the period from 1 January 2020 to 1 March 2025. The data, downloaded from the WAHIS webpage, were cumulated by the number of cases over the entire study period and categorized by animal types (domestic and wild). All the cartographic data were obtained using the QGIS software (v. 3.34.10). For territorial units, the nomenclature of territorial units for statistics (NUTS) utilized were sourced from https://gisco-services.ec.europa.eu/distribution/v2/nuts/ (accessed on 1 March 2025).

## 5. Host Reservoirs

The most susceptible species are in the Passeriformes order, which develop high viremia and excrete the virus in large quantities through oral and cloacal secretions. Other susceptible orders include Charadriiformes and Anseriformes, while Psittaciformes and Galliformes are less vulnerable [76]. Young animals are more susceptible to infection than adults, as demonstrated by an outbreak in Manitoba, Canada, where 25% of six-week-old geese exhibited illness and mortality. At the same time, birds aged 15 weeks to five years only seroconverted without clinical signs [101]. In Europe, the virus has been isolated in many wild bird species, both terrestrial and aquatic. Antibodies against WNV have also been detected in Europe’s colonial, marsh, and terrestrial bird blood. A broad spectrum of vertebrates is susceptible to WNV infection [84]. The virus or antibodies have been detected in numerous vertebrates, including alligators, alpacas, baboons, cattle, camels, dogs, goats, horses, crocodiles, cats, iguanas, lemurs, hares, wolves, macaques, pigs, skunks, bears, sheep, bats, raccoons, reindeer, rodents, turtles, mice, and humans [31,102,103]. The seroprevalence is higher in vertebrates in areas with long-standing viral circulation; for example, in South Africa, dogs showed a seroprevalence ranging from 8% to 37%, compared to only 2.4% and 5% in dogs from Missouri and New York, respectively, where WNV was more recently detected. Unlike birds, mammals play a minor role in virus transmission, as the viremia level is generally too low to infect vectors. WNV has also been identified in frogs (*Rana ridibunda*) in Tajikistan [104]. In the USA, WNV has infected captive alligators and crocodiles. The role of amphibians and reptiles in WNV transmission in wetland ecosystems remains unclear. Although alligators do not serve as reservoirs, they are important viral amplifiers due to viremia lasting up to 14 days [89]. Some bird species can become ill and show clinical signs, while others may become infected but show no signs of disease [105]. While house sparrows and crows are highly susceptible to WNV, they represent only a minor fraction of the analyzed mosquito blood meals and may play a limited role in transmission. The primary host species for maintaining and transmitting WNV in the USA is the American robin, mainly due to its attraction to dominant viral vectors [38]. Laboratory studies have confirmed that bird-to-bird transmission occurs, with various species demonstrating the ability for contact transmission [106]. The role of mammals as hosts could change; however, in the case of Aedes mosquitoes, which primarily feed on humans, they become primary transmission vectors for WNV [38]. Numerous studies have demonstrated that various animal species, such as Indian elephants (*Elephas maximus indicus*), Indian rhinoceroses (*Rhinoceros unicornis*), ring-tailed lemurs (*Lemur catta*), red pandas (*Ailurus fulgens fulgens*), snow leopards (*Panthera uncia*), and babirusas (*Babyrousa babyrousa*), are susceptible to WNV infection [10,12]. Severe WNV disease has been diagnosed in a wide range of avian species, including chukar partridges (*Alectoris chukar*), domestic geese (*Anser anser* domesticus), domestic Impeyan pheasants (*Lophophorus impeyanus*), owls (*Strigiformes*), pigeons (*Columbiformes*), vultures (*Cathartidae*), crows (*Corvidae*), cranes (*Gruidae*), pelicans (*Pelicanidae*), turtle doves (*Streptopelia turtur*), bald eagles (*Haliaeetus leucocephalus*), snowy owls (*Nyctea scandiaca*), flamingos (*Phoenicopterus* spp.), cormorants (*Phalacrocorax* spp.), and American crows (*Corvus brachyrhynchos*) [38]. Investigations during the WNV outbreak between 1999 and 2001 in the USA revealed that Corvus species are the most susceptible to the disease and the main amplifiers. Additionally, experimental studies following the 1999 WNV outbreak in American alligators in the Americas and WNV-associated “pix” lesions in saltwater crocodiles in Australia suggested that American alligators and saltwater crocodiles are also capable amplifiers of WNV, with high enough titers in their blood to potentially transmit the virus to mosquitoes [107]. Raccoons (*Procyon lotor*) were once thought to be potential reservoirs and amplifiers of WNV in Europe, but this hypothesis remains controversial and requires further study [108,109]. Seroprevalence studies of WNV in raccoons in the USA have reported a seroprevalence ranging between 34% and 54% [110,111]. Viremia and virus-shedding profiles in experimentally infected fox squirrels (*Sciurus niger*) have demonstrated their ability to maintain WNV infection and spread it to final hosts [12].

Many vertebrates, including various wild animals like squirrels, chipmunks, house mice, hamsters, bats, bears, wolves, tigers, lions, civets, striped skunks, raccoons, and crocodiles, are naturally exposed to WNV [79]. Additionally, mammals that frequently come into contact with humans, such as dogs, cats, sheep, pigs, and cows, are also affected [105]. Despite this, the exact role of these mammals in sustaining the WNV cycle in nature is still unclear [105,109].

## 6. Pathogenesis

WNV can infect the animal species belonging to the classes: Mammalia (land mammals, flying mammals, and cetaceans), Aves, and Reptilia [112,113].

For more than two decades many studies have been performed to clarify and understand the pathogenetic mechanisms as well as the roles played by WNV pathogenetic strains [114] in infected cells, in tissue/organ spread, especially in neuroinvasion. WNV shows many tropisms but the most important is for the central nervous system (CNS). In the CNS, a widespread neuroinvasion is recorded in WNV sensitive animals, in most species belonging to the aforementioned classes [12].

### 6.1. Sequence of Cellular Invasion Steps

In vivo, the first pathogenic step begins in keratinocytes and in immunoreactive cells, the Langerhans cells (LCs), belonging to the dendritic cell system. Both cells, keratinocytes and LCs, are infected after inoculation of WNV by blood-eating insects through infected saliva. In mice, inoculation of WNV mixed with blood-sucking ectoparasite saliva gland extract (SGE) showed a boost effect on viremia [115]. WNV replicates and after viremia spreads in the host tissues [105,116,117]. Granulocyte neutrophils and fibroblasts are also among the cells sensitive to WNV infection [105,118].

### 6.2. Cellular Invasion and Replication—The Keratinocyte and Langerhans Cell Model

Biological WNV transmission in the susceptible host is possible though the intradermal route by hematophagous insects, with inoculation of WNV–saliva during the dermal blood meal. WNV replicates in keratinocytes, which represent the target cell that plays an important role in the pathogenesis and persistence of intracellular infection, even for months, depending on the animal species: four months in mice, one month in avian species, six days in human keratinocyte cell cultures. In LCs, WNV replication begins between one and three days post-infection (dpi) [119] and later infected LCs move, through lymph drainage, to regional lymph nodes for antigen-presentation. WNV infection spreads systemically in the host. WNV skin infection occurs without inflammation as a host unspecific response; this condition could prevent skin viral clearance [117,120,121,122]. Upon skin infection, WNV persists inside keratinocytes during their biological cycle without keratinocyte-to-keratinocyte viral spread [117].

### 6.3. WNV Entry, Replication, Translation, and Viral Particle Assembly

For WNV, like for all Orthoflaviviruses, cell infection is carried out by endocytosis, and afterwards, viral particles are transported, wrapped in endoplasmic vesicles, from the entry point, the cell membrane, to the endoplasmic reticulum (ER), in which virus biogenesis begins. In the ER-associated membrane, the generation of the RNA template takes place for replication and protein translation. Immature viral particles mature in the Golgi network and are then released by exocytosis [123,124].

### 6.4. WNV Entry Routes, Neuroinvasion, and Cellular Lesions

WNV neurotropism is the cause of the neuro-clinical disease and neuropathology damages, mainly in humans and horses [12]. Among CNS infection routes, at least four are supposed: (1) Neuroinvasion carried out by viral transcytosis of endothelial cells based on the interaction of WNV, directly or carried out into infected cells, with the endothelial cells of the blood–brain barrier (BBB). WNV can cross the BBB, inducing an alteration of intercellular adhesion molecules (e.g., ICAM-1) and matrix metalloproteinase-9, followed by a change in the selective semipermeable endothelial barrier as well as of the immune responses to defend the CNS from pathogen invasion; (2) centripetal neuroinvasion through peripheral nerves or olfactory neuron infection (airborne WNV infection); (3) “Trojan-horse mechanism”, with CNS infiltration of infected immune cells through the circulation in the cerebro-spinal fluid (CSF); (4) retrograde axonal transport to CNS from WNV-infected peripheral neurons [41,105,125,126,127].

Degenerated neurons are detected after WNV infection of the CNS as well as the involvement of the immune response. In experimental murine infection models, neuronal degeneration of neurons of the ventral horn of the spinal cord are observed. Leukocytes infiltration is also reported [128].

Conflicting studies do not allow for affirming the role of autophagy as a cause of neuronal death [129], while apoptosis and pyroptosis induce neuronal death during WNV infection [127,130,131].

A study was performed on two WNV strains, a non-neuropathogenic (WNV-MAD78) and a neuropathogenic (WNV-NY), grown on human endothelial cells and astrocyte cell cultures. Astrocyte cell cultures have an important role in WNV replication. The non-neuropathogenic WNV strain showed delayed and reduced viral replication in comparison to the neuropathogenic strain (WNV-NY).

Based on the experimental results, the crucial role of astrocytes in CNS infection and neuroinvasion should be considered [114]. An in vitro study performed on a murine cell culture had also pointed out the role played by astrocytes in the persistence of WNV infection in the CNS [132].

## 7. Clinical Signs

WND is an infectious condition caused by a Orthoflavivirus belonging to the Flaviviridae family. It is transmitted primarily by mosquitoes of the genus *Culex* spp., which naturally infect avian hosts. Occasionally, other vertebrates, including humans and horses, can become infected; however, these are considered dead-end hosts as they do not develop sufficient viremia to re-infect mosquitoes [106]. Birds, in contrast, exhibit viremia levels high enough to facilitate mosquito reinfection, thereby sustaining the transmission cycle [133].

Transmission occurs through the blood meals of mosquitoes feeding on an infected host. Birds play a key role in maintaining the infection cycle, as they exhibit high levels of viremia, enabling them to infect new mosquitoes. Mosquitoes of the genus *Culex* spp. are the primary vectors, although other genera may also contribute to transmission. Humans and horses, on the other hand, do not develop significant viremia and are therefore considered dead-end hosts. In humans, viremia levels ranging from 0.06 to 0.5 PFU/mL have been reported, while experimentally infected horses have shown viremia levels ranging from 10 to 1000 PFU/mL at most [134].

The disease primarily affects birds, followed by humans and horses.

In humans, the disease is often asymptomatic, with only 21–26% of infected individuals developing self-limiting clinical symptoms. Of these, only 5% receive a diagnosis of WND [135]. Severe neuroinvasive forms develop in approximately 1% of cases [136].

Similarly, in horses, the disease is often asymptomatic, with 10–39% of infected animals showing clinical signs [137]. However, subclinical forms are likely under-reported in veterinary medicine due to the absence of significant medical intervention and the animal inability to express mild discomfort. The neuroinvasive rate in horses is higher than in humans, ranging from 8 to 10% [134].

Birds, particularly corvids, play a crucial role in the disease’s epidemiology. They are the most susceptible species and exhibit symptoms due to the infection of multiple organs, including the liver, spleen, kidneys, heart, and especially the CNS. Affected birds typically succumb within 24–48 h [138].

In general, clinical manifestations in humans and horses can be categorized as asymptomatic, symptomatic without neuro-invasiveness (primarily characterized by fever), and symptomatic with neuro-invasiveness, which includes conditions such as meningitis, encephalitis, and poliomyelitis-like syndromes.

### 7.1. In Humans

In humans, the most common symptoms of the non-neuroinvasive form include fatigue (96%), fever (81%), headache (71%), muscle pain and weakness, vomiting, diarrhea, and sensitivity to light. The most characteristic sign remains fever, which persists for 5 or more days, and typically, fatigue and muscle weakness continue for over 30 days even after the fever has subsided. In less than 1% of cases, individuals develop neuroinvasive forms, characterized by encephalitis, meningitis, and poliomyelitis-like syndromes with flaccid paralysis, associated with inflammation of the spinal cord and brain.

Findings at clinical examination in patients with neuroinvasive forms include tremors (35%), cognitive difficulties (45%), cranial neuropathy including facial weakness (16%), and coma (25%). Ataxia with generalized weakness (74%), asymmetric gait (15%), upper limb weakness (7%), and paraparesis (4%) are commonly reported [139]. Ocular manifestations, including blindness, chorioretinitis, anterior uveitis, and retinal hemorrhage, are well-documented in humans [140]. Additionally, some rarer clinical manifestations with causal links to WNV infection, less known, include rhabdomyolysis, hepatitis, pancreatitis, myocarditis, cardiac arrhythmias, and renal failure. Among individuals who develop neuroinvasive disease, the reported mortality rate ranges from 10% to 30%, depending on age and immune status. Approximately 60% of patients hospitalized with neurological forms show clinical signs for more than 90 days after diagnosis [139].

In a case report published in 2023, a 50-year-old woman, hospitalized with clinical symptoms of neuro-invasiveness, showed regression of trunk and head tremors after 12 months of treatment, although ataxic gait and hand tremors persisted, likely due to cerebellar atrophy. Brain MRI revealed clear involvement of the posterior thalamus and much of the midbrain. Parkinsonian signs (rigidity, bradykinesia, instability) and cerebellar signs (dysmetria, nystagmus, ataxic gait) were also reported, found in about one-third of individuals with neuroinvasive forms of WND [141].

Neuroinvasive forms are characterized by three types of mechanisms: (1) inflammatory processes causing acute encephalomyelitis due to immune-mediated disorders, with predominant effects on the white matter of the brain and spinal cord; (2) toxic-metabolic processes associated with secondary cardiac, pulmonary, or hepatic disorders, leading to hypoxic–ischemic lesions primarily affecting cortical gray matter and the upper portion of the cerebellar cortex; (3) vascular processes leading to multifocal cerebral arthropathy, causing reversible encephalitis [142].

### 7.2. In Horses

In horses, clinical symptoms are comparable to those in humans, but pauci-symptomatic forms are rarely observed following natural infections. Some horses develop only mild lethargy with a short-duration fever, and these cases are typically unrecorded. Along with signs such as weakness, anorexia, loss of appetite, and lethargy, ocular conditions have been reported, though blindness is considered one of the rarest clinical signs. More commonly, the following symptoms can be observed: enterocolitis, colic, rectal prolapse, lameness, which can present in various forms, including laminitis, cervical and thoracic pain, anemia, inflammation of the tongue, jaundice (indicating hepatic involvement), and urinary dysfunction (indicating renal involvement) [143]. Colic-like behavior often appears first and persists for a brief period before severe neurological disorders occur; it can be a behavioral disturbance indicating CNS involvement but may also present as true colic with abdominal pain, caused by damage to the autonomic nervous system.

Neuroinvasive forms experimentally manifest approximately 7 dpi and are characterized by ataxia (75%), muscle weakness (55%), fever, anorexia, lethargy, hyperesthesia, teeth grinding, hydrophobia, anxiety, circling movements, generalized muscle tremors, nystagmus, cranial nerve deficits, and facial paralysis. While both lineage 1 and 2 cause gait incoordination, lineage 2 more commonly causes ataxia. Ataxia can affect both the forelimbs and hindlimbs and may be notably asymmetric, potentially affecting all four limbs. Both weakness and paresis are characteristic neurological signs of brain and spinal cord involvement; weakness often progresses to recumbency in approximately one-third of cases [144].

Similarly to what is observed in humans, the WNV in horses exhibits a predilection for the motoneurons of the cranial nerves in the mesencephalon and rhombencephalon, leading to clinical signs such as difficulty swallowing, drooling, and unilateral facial paralysis [145]. In equines, the spinal cord and the gray matter of the mesencephalon and rhombencephalon are most affected, while the cerebral cortex appears to be less involved. CNS lesions observed in horses include mild to moderate meningeal hyperemia, subdural exudates, and focal areas of hemorrhage within the brainstem and spinal cord. Spinal cord lesions are restricted to the gray matter of the ventral and lateral horns in horses, and the more anterior horns in humans. Histologically, the lesions resemble those found in humans with polioencephalomyelitis, characterized by lymphocytes, a small number of macrophages, and granulocyte neutrophils infiltration [146].

As observed in humans, long-term effects are seen due to prolonged inflammation, which leads to neuron damage and dysfunction, with apoptosis of the neurons and a decrease in neurogenesis. This may help explain why the long-term effects of the infection result in cognitive impairment that mimics neurodegenerative diseases in humans, such as Alzheimer’s and Parkinson’s diseases [147].

The mortality rates of equines that develop neuroinvasive disease range from 22% to 36%, with higher rates in older and immunocompromised horses, as well as in foals under 12 months of age. Horses that do not experience appetite loss and receive adequate support, including being fed while lying down, have better chances of survival [148].

## 8. Genetic Susceptibility to Develop Neuroinvasive Forms

The incidence of neurological forms increases with age and includes encephalitis, meningitis, acute flaccid paralysis, peripheral neuropathies, polyradiculoneuritis, optic neuritis, and acute demyelinating encephalitis. CNS damage is due to several factors: direct insult, cytotoxic immune response, perivascular inflammation, and the formation of microglial nodules.

While it is well-established that the greater predisposition to develop severe neuroinvasive forms increases with age and in immunocompromised individuals, there is strong rationale to believe that there is a genetic predisposition to develop such severe forms of the disease [149].

In horses, a higher incidence of neurological forms has been recognized in certain breeds and herds where previous cases were reported. In this species, increased susceptibility to encephalitis is associated with a genetic variation in a gene that codes for 2′-5′-oligoadenylate synthetase 1 (OAS1). This protein is involved in the molecular pathway of ribonuclease-L (RNase-L) activation, which degrades viral RNA [150].

A study also found a correlation between specific genotypes and immune-related genes, such as those encoding for mitochondrial antiviral-signaling (MAVS) protein, natural cytotoxicity receptor 2 (NCR2), and interleukin 10 (IL-10) [151].

In humans, a significant genetic variability has been linked to neuroinvasive disease. Several polymorphisms of the HECT and RLD domain containing E3 ubiquitin protein ligase 5 (HERC5) gene have been associated with neuro-invasiveness. Although the antiviral mechanism is not yet fully understood, it has been proved that HERC5 exhibits broad antiviral activity, including the inhibition of immunodeficiency virus type 1 (HIV-1) assembly, attenuation of influenza A viruses (IAV), and reduction in the impact of human papillomaviruses (HPV) [152,153].

Genes encoding for the human replication factor C (RFC), also known as activator-1, also play an important role. RFC is a primer recognition protein composed of five distinct subunits and enables effective DNA elongation in the presence of single-stranded DNA-binding proteins. RFC recognizes 5′ phosphate termini of double-stranded telomeric repeats and is likely involved in the stability and turnover of telomeres, potentially facilitating viral replication [154].

Consistent with the findings in horses, it has been shown that variability in the OAS gene is associated with encephalitis and flaccid paralysis. Symptomatic infection is also linked to several variations in the interferon regulatory factor 3 (IRF3) gene (a regulatory factor of interferon), and Myxovirus resistance 1 (Mx1) which encodes for an IFN-inducible guanosine triphosphate protease (GTPase) with antiviral gatekeeper properties [150].

We are still far from fully understanding all the genetic mechanisms that increase an individual’s susceptibility to developing neuroinvasive forms of WND, but there are strong reasons to direct research in this direction.

## 9. Immune Responses to WNV

The evaluation of immunity to WNV includes the immune response in birds (main reservoir of WNV), the immune response in mosquitoes (primarily of *Culex* spp.) that become infected after feeding infected birds and represent the main vector for transmission, and the immune responses in receptive mammals, such as horses and humans.

### 9.1. Birds

In different permissive bird species, WNV can replicate at high levels. After infection, WNV stimulates innate pathogen-associated molecular patterns (PAMPs), such as the OAS pathway through which double-stranded viral RNA (dsRNA) is recognized by the OAS protein. In turn, 2′-5′-oligoadenylates bind the (RNase-L) enzyme, which cleaves viral RNA; the signaling cascade eventually induces programmed cell death (i.e., apoptosis) of infected cells [52].

In domestic chickens and different wild bird species, it was observed that humoral immunity in terms of an antibody response is mounted between 5 and 10 dpi, when viremia decreased and clinical signs can be observed; serum antibodies can be detectable for several months [155]. Neutralizing antibodies (NAs) and hemagglutinin-inhibiting (HI) antibodies rise concomitantly and can last even for years. These antibodies, both IgM and IgG isotypes, are pivotal for an effective humoral response and to confer long-lasting protection [155,156,157].

Maternally derived antibodies (MDAs) can be transmitted as passive immunity and proved to be neutralizing even though the levels decrease over time [52]. However, these antibodies are important for early protection in the newborns. Also, cross-protection against different types of Orthoflaviviruses and different strains of WNV has been observed in wild birds.

Experimental trials have shown that antigen-specific T cells are induced post-vaccination, as observed in mouse models, and are thought to be important for efficacious protection [138].

In infected birds, hematopoietic and mononuclear/phagocytic cells may be involved in the pathogenesis, as the virus can replicate in these cells and spread to different tissues. Viral proteins are detected in dendritic cells (DCs) and macrophages in the spleen, dermis, and subcutis. Highly susceptible avian species can have high viremia and viral spreading into the main organs, with minimal inflammation and no neurological involvement, while fewer receptive avian hosts that can survive longer may show chronic reactivity and lesions in the CNS as WNV can cross the BBB [158].

*Culex* spp. mosquitos are the main transmission vector for WNV from and to infected birds (reservoir). It was observed that other species, such as *Aedes* spp. Mosquitos, are permissive for infection, and transmission can be achieved through ticks in domestic birds [155].

### 9.2. Mosquitos

Immunity in mosquitos is composed of innate immune responses which are responsible for arthropod survival. However, WNV establishes a persistent infection in mosquitoes both in vitro and in vivo which allows the virus to persist in nature and spread to other permissive hosts far from the initial infection area. After ingestion of the virus through a blood meal, WNV reaches and interacts with pathogen recognition receptors (PRRs) in the epithelial cells of the midgut. Afterwards, the virus enters by endocytosis and replicates in the permissive cells and spreads through the hemolymph to the target organs, including the salivary glands. The mosquito counteracts viral infection through an antiviral immune response mainly consisting in the following: (1) the innate immune pathway and (2) the RNA interference (RNAi) pathway [38]. The innate response acts through the Toll-, Janus kinase/signal transducer and activator of transcription (JAK-STAT)-, and immune deficiency (IMD)-conserved pathways. These pathways are activated through the recognition of PAMPs, and then the signal is transduced to the activation of the NF-kB transcription factor REL-2 for the production of antimicrobial peptides (AMPs) such as defensins [159,160,161].

The RNAi pathway, including the P-element induced wimpy (PIWI)-interacting RNA (piRNA) pathway, acts as a sensor induced by dsRNA, which is a duplex form of WNV genome inside the cells during the virus life cycle. In this pathway, the RNase-3 enzyme Dicer-2 (Dcr-2) activates REL-2, which in turn up-regulates the expression and secretion of Vago (an antiviral cytokine-like molecule, acting like interferon in mammals), resulting in the activation of the JAK/STAT pathways and antiviral genes [160]. In response to this antiviral pathway, WNV (e.g., WNV_KUN_ strain) generates viral non-coding subgenomic flavivirus RNA (sfRNA), which is able to interact with Dicer-2 and to resist to degradation [52].

In addition, the midgut cells produce a complex matrix composed of chitin, glycoproteins, and proteoglycans to dampen viral replication [162]. The cells in the midgut and salivary glands can also undergo apoptosis to limit WNV spread in the mosquito tissues and organs [159].

### 9.3. Horses

Horses are also susceptible animals to WNV infection; similar to humans, horses mount effective innate and adaptive immune responses to WNV to mediate viral clearance. Increased levels of interferons type 1 (IFN-alpha and IFN-beta), type 2 (IFN-gamma), and nitric oxide (NO) are observed at early phases of infection in peripheral blood lymphocytes and monocytes, lymph nodes and spleen. The innate response also relies on an IFN-dependent OAS-1 response and cytokines such as IFN-alpha, CXC chemokine ligand 10 (CXCL10), toll-like receptor 3 (TLR-3), interferon-stimulated gene-15 (ISG15) and IRF-7 in the brain. The immune response detected in the CNS include interleukin (IL)-15 and IL-22, mitogen-activated protein kinase (MAPK), AMP-activated protein kinase (AMPK), the JAK/STAT-signaling pathway, and B and T cells. Macrophages and neutrophils were found to internalize viral antigen in the CNS [150].

Also the apoptotic phosphatidylinositol 3-kinase (PI3K)-mediated pathway is involved in the antiviral response [52,163].

Immune-related genes such as mitochondrial antiviral-signaling protein (MAVS), natural cytotoxicity triggering receptor 2 (NCR2), and IL-10 seem to be involved in the WNV onset of encephalitis [150].

The adaptive immune response is mediated by antibodies, which can be vertically transmitted as MDAs from the mothers [163]. Virus-specific IgM peak within 2 weeks post-infection and decline afterwards, while IgG1 increase especially during secondary infections [164]. Variable virus-neutralizing antibody (VNA) levels were also detected upon experimental infection. Infiltration of CD4+ and CD8+ T cells was observed in the CNS of infected horses [150].

### 9.4. Humans

Humans have been studied less extensively regarding the molecular mechanisms of the immune responses to WNV because of practical reasons related to the replication cycle of the virus in nature in human hosts and the safety measures required for manipulating the virus in the laboratory. Therefore, the immune responses were studied starting from the information obtained in laboratory mice or non-human primates (NHPs), human cells cultivated in vitro or collected from peripheral blood mononuclear cells (PBMCs) of infected-asymptomatic or infected-symptomatic patients, and in convalescent individuals who recovered from WNV infection [52,165,166,167].

The human immune response to WNV infection relies on both innate and adaptive mechanisms which act synergistically to restrict virus diffusion in tissues and organs and mediate viral clearance.

The innate responses are characterized mainly by (1) the involvement of IFN-I and ISGs to induce an anti-viral state, (2) an OAS1/RNA-L pathway, (3) a non-traditional OAS1 pathway involving interaction with AU-rich elements (AREs) to sustain IFN-beta expression [168,169] and (4) apoptosis of infected cells (especially skin epithelial and keratinocytes) as rapid cellular response to infection [52].

TLR3, Mx protein, interferon-induced transmembrane protein 27 (IFI27), IRF-3, and tumor necrosis factor (TNF)-alpha are involved in prevention of clinical signs of WND [166,167].

Also, invariant natural killer T (iNKT) cells seem to be involved in early activation against the virus after WNV infects DCs and keratinocytes in the skin due to the mosquito blood meal. However, iNKT cells seem to be sub-optimally activated due to altered communication and cytokine secretion by DCs [170]. Absent in melanoma-2 (AIM2) protein, which is a cytoplasmic PRR, was found to be up-regulated in primary macrophages early after WNV infection [166,168].

Increased polymorphonuclear (PMN) cells and monocytes were detected during acute infection together with increased cytokines (TNF-alpha, IL-8, IFN-gamma) and the phosphorylated transcription factors pSTAT3 and pSTAT5. Higher expression of genes associated with anti-inflammatory CD16+ monocytes were detected in infected–asymptomatic subjects [167].

In elderly and immunocompromised individuals, these responses may be downregulated or dysregulated, and this can promote longer viral detection in the host tissues and organs and/or the onset of neurological signs due to the virus crossing of the BBB [165]. It was also observed that WNV-infected cells can communicate through the secretion of small extracellular vesicles (EVs), such as exosomes, which incorporate RNA molecules such as messenger RNA (mRNAs), micro RNA (miRNAs), and small non-coding RNA (sncRNAs) that are able to regulate inflammation and immunomodulation [135,171,172].

It was demonstrated that a high fraction of severely affected WND patients developed auto-antibodies able to neutralize the IFN-alpha and IFN-omega innate responses [55,56]. These interferons are secreted into the blood especially by plasmacytoid DCs (pDCs) and may counteract the spread to the brain and the onset of neurological signs. Therefore, elderly individuals and those affected by autoimmune conditions may be at more risk of neurological signs if they become infected by WNV.

It was demonstrated that IL1-beta (IL1β) and chemokines/chemokine receptors, such as C-C motif chemokine ligand 2, CCL2 (i.e., monocyte chemoattractant protein 1, MCP-1), CCL5, CXCL10, and C-C motif chemokine receptor type 5 (CCR5, i.e., CD195), are produced in the CNS and are associated with severe neurological symptoms [166,173]. Also, IL6 and TNF-alpha over-production was demonstrated to be pivotal for WNV-induced prolonged systemic inflammation and neuroinflammation, leading to WNND [173].

Regarding the adaptive immune system, virus-specific B cells and T cells play an essential role in the clearance of WNV infection.

IgM is the first antibody isotype produced upon primary infection and is of great importance during the early humoral immune response, while an IgG response increases later and then remains constant or declines in WNV-infected seropositive subjects. Serum IgM persistence was detected in infected individuals, thus suggesting a prolonged antigen presence and stimulation [165].

Cytotoxic T cells (CD8+) are able to proliferate and kill infected cells upon recognition of the WNV antigen presented on the major histocompatibility complex 1 (MHC I). The presence of CD8+ T cells in the CNS has been shown to be essential for viral clearance. In addition, for a sufficient specific humoral response, CD4+ T helper 1 (Th1) cells are required. The clonal expansion of effector memory re-expressing CD45RA (T_EMRA_) T cells is involved in later responses during the first year [167].

MHC II deficiencies lead to increased mortality and increased viral persistence in the CNS. Symptomatic patients and patients with chronic infection showed higher expression of the inhibitory receptors T-cell immunoglobulin and mucin domain 2 (Tim2) and programmed cell death protein 1 (PD-1, i.e., CD279) on both CD4+ and CD8+ T cells. WNV can in fact persist in the host inside different types of cells together with high levels of inflammatory cytokines in the blood [165,166]. Individuals with hematological and T cell abnormalities are at higher risk of developing WNND.

Also, T regulatory lymphocytes (Tregs) are involved in the cellular response in that subjects with a higher circulating cell fraction and higher levels of IL-10 showed no symptoms or less severe disease; this highlights the fact that a regulated adaptive cellular response is very important for the outcome of WNV infection [165,167].

## 10. Clinical Pathology and Imaging

Clinical pathology plays a crucial role in the knowledge and management of WNV infection in both human patients and horses. Hematological tests reveal that anemia (41.1%), as a moderate reduction in circulating hemoglobin, is very common in patients with WNF. Moreover, both leukocytosis (35.9%) and leukopenia (8.6%) can be observed, as well as thrombocytopenia (14.9%) and hyponatremia (33–50%) [174,175,176,177]. CSF examination shows severe pleocytosis (usually less than 500 cells/µL) in patients with meningitis (97%), encephalitis (95%), and acute flaccid paralysis [177,178]. While pleocytosis is usually lymphocytic, typical of viral meningitis, CSF obtained in the early stages of the disease reveals a neutrophilic predominance [178]. Protein levels are increased in patients with acute flaccid paralysis and, conversely, appear reduced in patients with encephalitis. Additionally, lymphocyte counts are increased in the CSF of patients with acute flaccid paralysis [144,178,179,180,181]. Neutrophilic pleocytosis is observed in patients with meningitis (45.4%) and encephalitis (36.9%) [182,183]. In an article analyzing CSF from 27 patients undergoing lumbar puncture, most showed increased protein levels (>45 mg/dL) in 82% of cases and pleocytosis in 92% (mostly lymphocytic—80%) with normal glucose levels in 85.2% of cases [184].

In general, MRIs are frequently normal, but signal abnormalities can be observed in the basal ganglia, thalamus, and brainstem in patients with encephalitis and in the anterior tract of the spinal cord in patients with poliomyelitis-like syndrome [179,185,186]. In general, abnormal findings may be observed several weeks after the onset of the disease [187,188,189]. In the same study by Moreno-Reina and co-workers in 2022, among the same patients, all of whom underwent computed tomography scan CTS, only two abnormalities are shown: in one case, bilateral symmetric hypodense areas at the mesial region of the temporal lobes, while in the other, the same hypodense areas were detected in the basal ganglia and thalamus, with greater involvement on the left side. In patients undergoing MRI, 10 showed alterations: in 44.4% (8 patients), one or more findings were compatible with meningoencephalitis-related edema (T2/Flair signal hyperintensity) in the brainstem (38.9%), mainly bilateral, involving the mesencephalic substantia nigra; thalamus (33.3%); basal ganglia (5.6%); cerebellum (5.6%); hemispheric white matter (5.6%); temporal lobes (5.6%). One scan revealed increased infratentorial leptomeningeal intensity after contrast administration, and another showed a restricted diffusion focus on the left thalamus. Spinal MRIs showed areas of increased intramedullary signal on T2/STIR from T8 to the conus medullaris, consistent with the degree of myelitis [184].

Other studies also highlight electroencephalogram (EEG) abnormalities, such as generalized slowing, often predominant anteriorly or temporally, and triphasic sharp waves [190,191]. These abnormalities remain non-specific [192].

Clinical pathology in equids has been studied both in the field, evaluating sick animals, where complete blood count (CBC) and biochemical tests often revealed no significant findings [59], and through processing samples from experimentally infected equines [143,193,194,195]. The most common hematological findings are lymphocyte count reduction and, though less frequent, neutrophilia [196]. The role of neutrophils in this context is unclear, but they may play both protective and damaging roles during infection [197]. Horses with severe disease show both monocytic and lymphocytic pleocytosis, with neutrophilic forms occasionally observed [144,180]. In eight naturally infected horses presenting with encephalomyelitis, differential leukocyte counts showed alterations compatible with both suppurative and non-suppurative infections and lymphocytic pleocytosis. In three horses, mild-to-moderate increases in total protein were observed [198]. In another study testing a horse with naturally occurring encephalomyelitis, low leukocyte counts in the CSF were reported, along with high protein levels and xanthochromia [143].

In a study, all ten goshawks (*Accipeter gentilis*) included in the research and testing positive were subjected to radiological examination, with nine undergoing computed tomography. The images revealed multiple tissue opacities in the pulmonary parenchyma or air sacs, with a diagnosis of pneumonia/air sacculitis in five out of ten. Splenomegaly was observed in two animals, as were trauma-induced injuries (fracture of the carina and scapula) in another two out of ten birds. From a hematological standpoint, elevated creatine kinase (CK), aspartate aminotransferase (AST), alanine aminotransferase (ALT), alkaline phosphatase (AP), and uric acid (UA) levels were detected. Furthermore, either moderate or severe anemia was noted in seven out of eight birds sampled [199].

For serological, virological, and molecular diagnostics, reference will be made to the dedicated section of this review.

## 11. Differential Diagnosis

The differential diagnosis among the various forms of encephalitis can be quite complex due to the overlap of symptoms. Bacterial, fungal, parasitic, protozoal, and autoimmune encephalitis can often be more easily distinguished with simpler laboratory tests. However, it is more challenging to differentiate other forms of viral encephalitis, which may show similar hemato-biochemical profiles and require more advanced laboratory examinations for differentiation. Viral infections are often clinically indistinguishable from WND, which are numerous.

### 11.1. Herpes Viruses

Herpesvirus encephalitis in humans and horses show symptoms very similar to those observed in WND. However, in humans, there is a greater involvement of cognitive functions compared to the motor impairments more commonly observed in WND. In horses, clinical signs can be differentiated in some cases, such as more pronounced lumbosacral signs in herpesvirus infections (e.g., ataxia of the hind limbs, weakness, bladder paralysis) that differ from those induced by brainstem damage in horses with WNV infection (e.g., ataxia in all four limbs, muscle fasciculations, hyperesthesia). Nevertheless, there can be overlap in the clinical presentation. Epidemiological characteristics, such as seasonality or comorbidity among horses in the same location, can also help differentiate between the two infections [200]. In both humans and horses, laboratory tests are crucial for proper diagnosis.

### 11.2. Varicella Zoster

Encephalitis during Varicella Zoster virus infection is secondary to inflammation of the cerebrovascular endothelial cells and is associated with acute focal deficits due to involvement of large vessels. Such lesions can be differentiated using advanced imaging diagnostics [201].

### 11.3. Measles

Measles can lead to neurological forms classified into four types: (1) acute encephalitis (fever, rash, cough, and rhinorrhea); (2) post-infectious encephalitis (within two weeks of the rash and characterized by altered mental status and seizures); (3) inclusion body encephalitis (behavioral changes, seizures, myoclonus, and coma); (4) subacute sclerosing pan-encephalitis (occurring up to eight years after infection, characterized by intellectual decline progressing to coma and then death).

### 11.4. Rabies

Characterized by nearly 100% mortality, rabies can be divided into two forms: encephalitic and paralytic. The encephalitic form begins with headache, fever, paresthesia, followed by neurological dysfunction and coma, leading to death; the paralytic form is characterized by weakness and flaccid paralysis, progressing to quadriplegia.

The differential diagnosis of rabies should be considered whenever neurological signs appear in areas where the disease is endemic or in individuals who have visited such regions.

### 11.5. Japanese Encephalitis

Japanese encephalitis (JE) is one of the most important viral encephalitis worldwide and has a significant impact especially in low- and middle-income countries with limited access to healthcare [202].

JE predominantly affects children, although older adults can also develop acute neuroinvasive infections [203]. The disease can also be observed in adults who have recently moved to areas where the virus is present or among unvaccinated travelers to endemic regions. While often asymptomatic, the mortality rate reaches approximately 30% when symptoms appear [203]. The causative agent of JE, the Japanese encephalitis virus (JEV), is a member of the Flaviviridae family and the Orthoflavivirus genus. Its transmission cycle is facilitated and promoted by vertebrate hosts and *Culex* spp. mosquitoes. Pigs and heron-like birds develop high-titer viremia and act as amplifying hosts for JEV. Although JEV does not cause encephalitis in adult pigs, it triggers reproductive disorders such as fetal death, abortion, and congenital malformations, leading to significant economic losses in the livestock industry. Given that there is no specific treatment for JE, vaccination remains the only effective means of controlling JEV infection in both humans and animals. The symptoms of JE primarily affect emotional, cognitive, and neurological functions [204].

### 11.6. Chikungunya Virus

Chikungunya virus (CHIKV) (genus Alphavirus, family Togaviridae) circulates between Aedes (Ae.) mosquitoes and NHPs. Epidemics occur when an anthropophilic or opportunistic mosquito (such as *Ae. aegypti* or *Ae. albopictus*) bridges the zoonotic and anthropophilic cycles. Autochthonous transmission in Europe primarily happens during periods of returning travelers from endemic regions. The symptoms are characterized by fever and severe polyarthralgia, sometimes complicated by neurological signs [205].

### 11.7. Dengue Virus

Dengue virus (DENV) (genus Orthoflavivirus, family Flaviviridae) is one of the most widespread arboviruses globally and is mainly transmitted through *Ae. aegypti* and *Ae. albopictus* mosquitoes during urban transmission cycles. The symptoms begin after an incubation period of 2–7 days, characterized by fever, headache, myalgia, rash, and, in severe cases, hemorrhagic fever, with or without associated neurological signs. Clinically, the disease is very difficult to distinguish from WND [205,206].

### 11.8. Zika Virus

Zika virus (ZIKV) (genus Orthoflavivirus, family Flaviviridae) was first isolated in 1947 in Uganda and has since spread to many regions. The virus is transmitted by Aedes mosquitoes and primarily infects humans [205].

### 11.9. Usutu Virus

Usutu virus (USUV) (genus Orthoflavivirus, family Flaviviridae) was first isolated in South Africa in 1959 and later spread to Europe. Birds, especially blackbirds, are the primary hosts of the virus.

These viral infections also share similar seasonal patterns and overlapping epidemiology, often linked to the presence of vector insects, which, due to climate change, have spread quite uniformly across various regions [205].

Various viral infections can present with symptoms like WND. Sometimes, factors such as the seasonality of certain infections, epidemic trends, or travel to areas where the disease is endemic can help, but often the differential diagnosis can be truly challenging without the use of specific diagnostic tests.

## 12. Laboratory Diagnosis of WNV Infection

Diagnosing WNV infection in the laboratory is crucial for verifying the virus presence and tracking its spread, especially in veterinary and human contexts. The diagnostic process typically involves serological tests and molecular methods that provide a comprehensive approach to identifying the infection [207,208].

### 12.1. Diagnostic Methods

In animals, as reported by the European Centre for Disease Prevention and Control (ECDC) [79], WNV is diagnosed by National Reference Laboratories (NRLs) in 21 out of 30 EEA countries, with additional contributions from regional laboratories in France, Germany, Italy, and Spain, as well as local laboratories in countries such as Greece, Luxembourg, and Slovakia. Private laboratories and other public institutions were reported as being involved in diagnostic efforts in Austria, Germany, and Slovakia. At the same time, Norway relied on the European Union Reference Laboratory in France or the WOAH WNV Reference Laboratory in Italy.

### 12.2. Serological Tests

The most commonly used serological methods to detect antibodies against WNV include enzyme-linked immunosorbent assays (ELISAs) and neutralization tests [83,207,209,210]. Distinguishing between WNV from other cross-reactive Orthoflaviviruses poses a challenge, especially when serological methods are employed. Serological tests are commonly used, such as the detection of IgM and IgG in the acute or convalescent phase of infection, respectively. ELISA is widely used for detecting IgM, which typically indicates a recent infection, and IgG, which suggests a past infection. Although commercial ELISAs are available and relatively quick and cost-effective, they lack specificity in differentiating between WNV and other Orthoflaviviruses due to significant serological cross-reactivity, which may lead to false positive results. Similarities in their antigenic structures cause this cross-reactivity. Plaque Reduction Neutralization Tests (PRNTs) are often used as a confirmation and titration method of WNV-specific NA (in serum or cerebrospinal fluid [CSF]). Due to cross-reactions among Orthoflaviviruses, other Orthoflaviviruses usually have to be tested in parallel. However, the specificity of detection can be confirmed by the demonstration of a 4-fold increase in NA titers, comparing acute and convalescent sera [211]. At the same time, the PRNT has disadvantages that cannot be ignored [208]; the test implementation and procedure are complex, labor-intensive, and require specific safety measures according to the World Health Organization (WHO) Laboratory Biosafety Manual [212].

### 12.3. Molecular Tests

RT-PCR is a molecular diagnostic tool that detects WNV RNA in blood, CSF, or tissue samples during the early stages of infection, particularly in cases of severe neurological symptoms such as encephalitis or meningitis [83,213]. This method is invaluable for detecting the virus during the acute phase of infection, though the detection window is relatively short, typically limited to the first few days after infection [83]. Real-time RT-PCR offers more comprehensive results by measuring the viral load, which is crucial for monitoring WNV in human and animal populations and mosquito vectors.

Viral isolation: Although not commonly employed, virus isolation is still an option for verifying WNV infection in specialized laboratories. This method involves cultivating the virus in cell cultures, confirming its presence through various techniques such as immunofluorescence. However, this method is time-consuming, requires specific safety measures according to the WHO Laboratory Biosafety Manual [212], and may not be as sensitive as molecular techniques, particularly in later stages of the disease [83,213].

### 12.4. Testing Strategies

The choice of diagnostic tests and the timing of sample collection for detecting WNV infections depend on the species being tested [213].

For avian species, virus detection can be achieved through RT-PCR. Whole blood is preferred with EDTA as an anticoagulant, as heparin should not be used due to its inhibitory effects on PCR assays. Oropharyngeal or cloacal swabs should ideally be placed in a small volume of transport medium or saline, though oral swabs may not be suitable for certain bird species like raptors. Fresh tissue samples, such as brain, heart, kidney, liver, spleen, and bone marrow, can also be used. Formalin-fixed tissues from the same sample set as fresh tissues are acceptable but require an additional surcharge due to the extraction process for formalin-fixed samples. Virus isolation serves as an alternative detection method and can be applied to all samples suitable for PCR, except formalin-fixed tissue samples. For antibody detection in avian species, serum is the preferred sample, although plasma may also be used; however, the presence of anticoagulants can interfere with accurate antibody titer determination. Paired samples are essential for accurately assessing recent infections or vaccinations. The PRNT is a traditional test used for detecting Orthoflavivirus antibodies, although it may show cross-reactivity with other potentially cross-reactive Orthoflaviviruses.

For mammals, reptiles, and amphibians, detection can also be achieved using RT-PCR. Fresh tissue samples, including the brain, spinal cord, and CSF, are commonly used. Formalin-fixed tissue samples from the same set as fresh tissue are also acceptable, though they require an additional surcharge for extraction. Equine whole blood can aid in WNV detection; however, the low viremia levels and brief duration might reduce diagnostic sensitivity. Therefore, timely collection is critical, as the virus may no longer be detectable when clinical signs appear. Samples of whole blood from mammals, reptiles, and amphibians are not typically used for detections because they exhibit low viremia levels during infection. Virus isolation is possible with all samples suitable for PCR, except for formalin-fixed tissues. For antibody detection in mammals, reptiles, and amphibians, serum is the preferred sample, but plasma may also be used, though anticoagulant toxicity can interfere with accurate titer determination. Paired samples are required to assess recent infections or vaccinations accurately. Testing options include the PRNT and the microplate serum neutralization (SN) assay, available for equine and canine species, providing titer values two to 4-fold higher than the PRNT assay and offering a more cost-effective option. However, samples with low titers (≤16) in the microplate assay should be confirmed using the PRNT. For equine species, antibody capture ELISA tests are available. The IgM ELISA detects early immune responses due to infection and can use serum, CSF, or plasma upon special request, but interpretation must consider the animal’s vaccination status. The IgM and IgG ELISA panel can define a horse’s infection or vaccination status. However, paired serum samples may be necessary to differentiate infection from vaccination in vaccinated horses.

The accurate detection of WNV is crucial, particularly for epidemiological studies and monitoring the spread of the disease. Accurate diagnosis of WNV infection requires a blend of serological, molecular, and virological techniques. Serological tests like ELISA and PRNT are crucial for confirming the immune response, whereas molecular methods such as RT-PCR and virus isolation play a vital role in detecting the virus during the acute phase. Given the possibility of unapparent infections, diagnostic protocols must integrate both clinical assessment and laboratory results, using a combination of tests to confirm infection and differentiate WNV from other Orthoflavivirus-related diseases [207,214].

## 13. Pathology Induced by WNV Infection

In most mammalians, especially in humans and horses, WNV infection is sub-clinical or febrile, non-life-threatening and without CNS lesions. This phenomenon could be explained by the presence in the host of an immune resistance developed by antigen-stimulated memory cells (antibodies or cell-mediated response) to limit and clear out the WNV infection or for an efficient pre-existing innate and adaptive immune response [193]. In the last sixty years after its recognition in Uganda in 1937, WNV has circulated at first in Africa, then moving to Middle East countries, Russia and Europe, as above cited, as classical sub-clinical or febrile forms. In the 1990s, with the recognition of a new strain of WNV (lineage 1) with characteristics of neurotropism and inducing neurological lesions, there was a significant change in the danger of WNV for humans and horses, despite its low neuropathological risk registered in 1% of infected humans [215]. The pathogenicity of WNV infection in birds is important for the increased susceptibility of avian species to infection than other animal species. Serological investigations performed in several years and in several Countries have recognized antibodies for WNV or its antigen in over 326 domestic, wildlife, or zoo species [46]. Raptors and corvids, especially crows, jays, and ravens, are very receptive to WNV infection, often with evidence of neuro-muscular signs and neuro-histological lesions [46].

## 14. Mammalia

### 14.1. Order: Primates

#### 14.1.1. Humans

WNV neuroinvasion occurs in less than 1% of infected individuals, and the most prominent lesions are inflammatory and neurodegenerative. Inflammation involves the meninges (encephalomeningitis and spinal meningitis), brain (encephalitis), and spinal cord (poliomyelitis), while neurodegeneration is recognizable in telencephalic neurons and in lower motoneurons [113]. Neuro-clinical symptoms depend on the location of the neuroanatomical damage. Histopathologic features are quite unspecific and common to many other viral CNS infections. They are consistent with perivascular lymphocyte cuffing, microglial nodular reaction, and neuronal death and loss [216,217,218,219]. A case of fatal hepatitis was observed in South Africa [220].

#### 14.1.2. Non-Human Primates (NHPs)

(a) Natural Infection

At the Toronto Zoo, a geriatric Barbary ape (*Macaca sylvanus*), WNV-naturally infected with clinical neurological signs of encephalitis, was euthanized. At gross pathology, no CNS lesion was recorded, while gingivitis and bilateral femoro-tibial osteoarthritis, which are lesions compatible with its advanced age, were recorded. A worsening muscular atrophy condition was also recognized [221]. At histopathology, a non-suppurative meningoencephalitis was observed. The most relevant features were a bilateral symmetrical rhombencephalic diffuse gliosis, multifocal microglial nodules, and perivascular lymphoplasmacytic cuffing. The cerebellum, the telencephalon, and the mesencephalon were damaged. Meninges were also infiltrated by mononuclear cells. Extra-neural histopathologic findings were hepatocellular atrophy and interstitial nephritis. Immunohistochemical tests for WNV antigen showed immunopositivity in neurons, especially Purkinje cells as well as glial cells inside or close foci of inflammation (diencephalon, mesencephalon, cerebellum). WNV antigen was not detected in structural cells of liver, lymph node, lung, and kidney [221].

Two different serological surveys were performed in an outdoor non-human primate breeding colony in south Florida [222] and non-human primate breeding colony in southern Louisiana [223].

In Homestead and LaBelle (Florida), 200 Rhesus macaques (*Macaca mulatta*), 212 Cynomolgus macaques (*Macaca fascicularis*), and 108 Hamadryas baboons (*Papio hamadryas hamadryas*) were blood-sampled for serological investigations for WNV antigens. The WNV serological investigation returned relevant data in the Baboons (29%), Rhesus (11%), and Cynomolgus (9%) macaques [222].

In St. Tammany Parish (Tulane National Primate Research Center—TNPRC), Louisiana, a total of 1692 NHPs were blood-sampled, from August to November 2002. The seroprevalence for WNV was equal to 36% with a percentage slightly higher than 39 in Rhesus macaques, where the gender seroprevalence was 42.2% in females and 34.5% in males [223]. Authors matched the seroprevalence data of TNPRC with national ones and pointed out that TNPCR data were comparable to WNV infection rates registered in bird populations in the USA after human epidemics of West Nile encephalitis, as well as recorded during a WNV outbreak in 1999 at the Bronx Zoo, NYC (USA), in which immunopositivity in homed birds, captive and wild, was detected at 34% [223]. In both serological surveys, no data were available concerning gross pathology and/or histopathology.

(b) Experimental Infection

An experimental infection of Rhesus macaques (*Macaca mulatta*) and Common marmosets (*Callithrix jacchus*) with a virulent European WNV strain (WNV-Ita09) was performed. Higher rates of viral replication and a wider tissue distribution of virus was detected in absence of neuro-clinical signs in both monkey species. At necropsy, many tissues were sampled for testing the presence of WNV RNA by using a diagnostic nested-RT-PCR assay, but no gross lesions were observed in splanchnic organs and CNS. Histological investigation was not performed [224].

Both species reacted to WNV infection by developing a cellular (NK cells) and humoral (IgM and IgG production) immune response. Rhesus macaques and Common marmoset are susceptible to WNV with an equipotential immune response, as observed in humans [224].

#### 14.1.3. Other Primates in Wildlife

An epidemiological investigation involving the identification of antibodies to WNV antigens in neotropical monkeys in wildlife or captive in Costa Rica was performed. Blood was collected from four species of native NHPs: Howler monkey (*Alouatta palliata*) in wildlife, White-face monkey (*Cebus imitator*) in wildlife, Spider monkey (*Ateles geoffroyi*) captive, and Squirrel monkey (*Saimiri oerstedii*) in wildlife. Monkeys were captured using chemical immobilization darts in respect of protocols established by the Institutional Committee for the Care and Use of Animals (“Comité Institucional para el Cuidado y Uso de los Animales”) of the University of Costa Rica. Serum samples were tested for specific WNV antigen. Antibodies against WNV in two Howler monkeys were detected [225]. No data were available concerning gross pathology or histopathology.

On many occasions, Malagasy lemurs (*Lemur catta*) were serologically screened for WNV and tested for susceptibility to WNV by experimental infection [226]. In the first serological survey, dated 1978–1980, WNV antigens were recognized in 39 out of 209 lemurs (*Lemur catta*) [227]. In lemurs (*Lemur catta*), WNV antigen positivity, equal to 32%, was recorded in a study dated 1982 [226]. In an experimental exposure of brown lemurs (*L. fulvus*) to WNV, infection showed a subclinical course [228]. No data were available concerning gross pathology or histopathology.

### 14.2. Order: Perissodactyla

#### Equine (Domestic)

WNV induces neuro-clinical disease (motor disorders, cranial nerve deficits, seizures, hyperesthesia) in nearly 20% of infected horses [144,194,229,230,231]. Gross CNS pathology is often not obvious or very unspecific as brain edema either meningeal suffusion, petechiae, or otherwise as perivascular hemorrhage [232,233]. Nervous lesions were detectable histologically in ventral horns of the thoracic and lumbar spinal cord segments and were typical of poliomyelitis [232]. The most relevant histopathologic features were perivascular hemorrhages and/or perivascular cellular cuffing, in which lymphocytes, macrophages, and neutrophilic granulocytes were the most prominent cell populations represented [232]. Foci of glial reaction and neuro-satellitosis were also observed [232]. Similar lesions were present in the midbrain and rhombencephalon, while the telencephalon was less damaged [144,232]. Myocarditis, hemorrhages in the renal parenchyma and in the spleen pulp were recorded as well as lymphopenia in germinative center of lymphoid organs [232,234]. Tber [233] also described pulmonary lesions, such as congestion and edema, probably secondary to neurogenic (e.g., meningoencephalitis) or cardiac (e.g., myocarditis) failure. Sequelae associated with WNV encephalomyelitis were septic tenosynovitis, staphylococcal cellulitis, muscle atrophy, corneal ulcers, and other damages. Sequelae were recorded as single nosological entities or even in associated forms [144]. A serological survey, for epidemiological study purposes, conducted in eight provinces of Turkey demonstrated positivity for WNV-NAs in 1 out of 40 (2.5%) donkeys and mules [234]. Venter et al. [235] have highlighted the risk of transmission of WNV from horse-to-human during necropsy of a horse with a neuro-clinical form; the maximum risk is related to the contact of the prosector with horse infected CNS.

### 14.3. Order: Arctiodactyla

#### 14.3.1. Ruminants (Domestic and Wildlife)

WNV infection was described in domestic and wild ruminants, but only in sheep (*Ovis aries*), Alpaca (*Vicungna pacos*) [236,237], and Reindeer (*Rangifer tarandus*) [238], the disease was clinically recognized and described, as well as gross pathology and histo-lesions.

WNV_KUN_ is a WNV sublineage, designated as 1b, endemic across areas of Australia, isolated in *Cx. annulirostris* mosquitoes, a native insect whose habitat consists of brackish zones. *Cx. annulirostris* is recognized in Australia, in South Pacific Islands (Fiji, Micronesia), in the Philippines, and in Indonesia. The first WNV isolation occurred in 1960 from *Cx. annulirostris* caught in north Queensland named Kunjin belonging from the Aboriginal community living on the Mitchell River [239].

In 1966, Spradbrow and Clark [240] performed an experimental infection: WNV_KUN_ MRM16 strain was intracerebrally inoculated in nine calves. At gross pathology in only one calf, CNS lesions were recorded and described as hyperemia of brain/meningeal bloody vessels associated with brain edema, while in other experimental calves, lesions were recognized as hyperemia of bloody vessels mucosae membranes of gastro-intestine and conduction system of respiratory apparatus (nasal cavity, trachea, and bronchi); catarrh mucous secretions were also recorded.

At histopathology, a non-suppurative encephalitis was recorded in brains of eight out of nine calves examined. Neuronal degeneration, microgliosis in white and gray matter, and perivascular lymphocytes cuffing were the most relevant microscopic features [240].

In sheep, WNV natural infection associated with neuro-clinical symptoms was observed many times. CNS gross pathology was not obvious, while pulmonary edema could be the most relevant lesion observed at necropsy. A non-suppurative meningoencephalitis and poliomyelitis, in some cases, were the most consistent histopathological features. Neuro-histopathology was characterized by lymphoplasmacytic infiltration and perivascular lymphoplasmacytic cuffing. Scattered or focal glial aggregates, associated with neuronal death and neuro-satellitosis, were other microscopic lesions recorded, while the presence of histiocytes and neutrophil granulocytes was sporadic. Cerebellum could also be affected by neurogliosis and necrosis of Purkinje cells. Cerebellar lesions affected both the white matter and the gray matter, with greater histo-lesivity recorded in the CNS gray component [241,242,243,244].

In Alpaca (*Vicungna pacos*), unspecific gross pathology and WND characteristic histopathological features were observed. Histologically, multifocal polioencephalomyelitis (rhombencephalon) with perivascular lymphocytic cuffing in the meninges as well as axonal spheroids were reported [237]. More histopathological features in rhombencephalon and in the spinal cord were neuronal necrosis, astrocytosis, and proliferation of resident microglia cells organized in nodule or scattered as rod-like cells in the oblongata [236].

In Reindeer (*Rangifer tarandus*), at necropsy, no gross pathology was observed in any of the Reindeer examined. The histopathological study focused on the lesions in the CNS and kidneys. In the CNS rhombencephalon, the cerebellum and spinal cord were the most damaged anatomical regions. The inflammation was the prominent host reaction characterized by lymphohistiocytic infiltration (encephalomyelitis), and the same cellular mononuclear infiltration was also recorded in the renal interstitium [238].

Epidemiological studies performed in South Africa detected the presence of WNV in the biological matrix in one North American imported White-tailed deer (*Odocoileus virginianus*), in one cow and in one goat [231]. WND was diagnosed in white-tailed deer in the USA as early as 2004 [245]. Gross pathology features were inapparent, while, at histopathology, lymphocyte and plasma cell infiltration was observed in a scattered way in the nervous tissue. A perivascular lymphoplasmacytic cuffing, around blood vessels in rhombencephalon was recognized. CNS hemorrhages and neuronophagia were also reported as well as spleen congestion. Palatine tonsils were characterized by lymphocyte depletion in the germinative lymphoid center [245]. During a sero-surveillance survey in Serbia (2010–2012 years), antibodies (ELISA) were detected in Roe deer (*Capreolus capreolus*) in 18.7% of sera tested [246]. No data were available concerning gross pathology or histopathology in Roe deer (*Capreolus capreolus*).

#### 14.3.2. Swine (Domestic and Wildlife)

(a) Natural Infection

WNV infection in European wild boar (*Sus scrofa*) was performed in central Italy (Latium region) and in central Spain (Doñana National Park) [247,248]. In Serbia, antibodies to WNV were quantified as 17.6% [246]. During a sero-surveillance survey in Serbia (2010–2012), antibodies (ELISA) were detected in pigs in 15.4% of sera tested [246]. No data were available concerning gross pathology or histopathology in wild boars and pigs.

(b) Experimental Infection

In pigs, two experimental WNV infections were performed [249,250]. Viremia was observed in pigs of both experimental conditions [249,250], but seroconversion was observed in only one of them [250]. No clinical evidence was recorded in any experiments focused to detect the susceptibility of swine to WNV [250]. In both above-cited experiments [249,250], no data concerning gross pathology or histopathology were available.

#### 14.3.3. Cetacea

An investigation on WNV was performed in a 14-year-old male killer whale (*Orcinus orca*) which suddenly died at a marine park in San Antonio, Texas, USA. At necropsy, in the brain and cerebellum, mild multifocal meningeal hyperemia and petechial parenchymal hemorrhages were recognized. Moreover, in the right accessory lung lobe, focal, local extensive, fibrosis was observed, in addition to other parenchymal lesions, such as hemorrhages, congestion, and edema. In the CNS, moderate multifocal subacute vasculitis associated with perivascular hemorrhages, and necrosis with lymphoplasmacytic vessel wall infiltration and non-suppurative meningoencephalitis were recorded in mesencephalon, rhombencephalon, and cerebellum. Glial nodules were observed scattered in the telencephalic white matter. Unspecific WNV histological lesions were recorded in the respiratory tract (parenchymal abscess and fibrosis) and gastric ulcer. A multi-organ congestion was also recorded in the spleen, lymph nodes, and kidneys [112].

A retrospective molecular genetic study (PCR and immunohistochemistry, IHC) on dolphin brains, with anonymous etiological meningoencephalitis, was performed on 19 free-ranging cetaceans stranded on the coast of the Canary Islands (Spain). The cohort examined consisted of 15 striped dolphins (*Stenella ceruloalba*), 2 common dolphins (*Delphinus delphis*), and 2 rough-legged dolphins (*Steno bredanensis*). No antigen attributable to WNV was detected in dolphin CNS or in visceral organs or tissues tested (skin, muscles, lungs, liver, kidney, spleen, lymph nodes) [251].

#### 14.3.4. Marine Mammals

In 2006, Del Piero and co-workers [252] published the first detailed study on WNV lesions in the harbor seal (*Phoca vitulina*), the first to report WND in marine mammals.

At necropsy, hyperemia of rhombencephalon and spinal cord was recorded and reduction in the thickness of body fat deposits was also observed; a non-suppurative encephalomyelitis mainly interesting the gray matter of the rhombencephalon and spinal cord were the most relevant CNS lesions. Symmetrical bilateral lesions were recorded in the spinal cord, in which the most prominent cellular changes observed were lymphocytes and plasma cells organized around the blood vessels (perivascular cuffing). Gliosis, nodular gliosis, axon swelling, of varying degrees of severity, were recorded in rhombencephalon, cerebellum, mesencephalon, diencephalon and in basal ganglia. Neuronal damage was detected in CNS regions. Mononuclear leptomengitis involved meninges covering the spinal cord and cerebellum. Microhemorrhages were also a feature affecting the cortex of cerebellum [252].

WNV infection in two harbor seals (*Phoca vitulina*) showed no lesions in one case (brain not submitted) and very basic lesions in the second case, referable to meningitis and myocarditis at histopathology [253]. A serosurvey for WNV was reported in marine mammals after the dead of four harbor seals with neurological signs with consistent CNS lesions supporting the presence of WNV-induced neuro-lesions. At CNS histopathology, the four harbor seals showed multifocal, non-suppurative encephalitis. A serological investigation was performed in birds and many animals, marine mammals included, of the Sea World San Antonio Theme Park, San Antonio, TX. Blood Samples were sent to the Texas Veterinary Medical Diagnostic Laboratory (TVMDL) and/or to Cornell University for titers.

Marine mammals with antibodies to WNV did not present neuro-clinical symptoms. The WNV-positive marine mammals tested were California sea lion (*Zalophus californianus*), harbor seal (*Phoca vitulina*), Hawaiian monk seal (*Monachus schauinslandi*), Warlus (*Odobenus rosmarus divergens*), Pacific white-sided dolphin (*Lagenorhynchus obliquidens*), Beluga whale (*Delphinapterus leucas*), and Common bottlenose dolphin (*Tursiops truncates*) [253].

#### 14.3.5. Flying Mammals—Chiroptera

##### Natural Exposure

Two healthy bats, brown (*Myotis lucifugus*) and northern long-eared (*Myotis septentrionalis*), tested positive for WNV-NAs in a study performed on bats in New Jersey and New York in 2002 [254]. No data were available concerning gross pathology or histopathology. Considering that in avian species the virus was isolated from the oral cavity [255], although not investigated in bats, the practice of grooming must be considered as an active tool for the spread of WNV within the bat community [254].

### 14.4. Order: Carnivora (Carnivores and Mesocarnivores; Domestic and Wildlife)

#### 14.4.1. Carnivores—Domestic

(a) Natural Infection

WNV was isolated for the first time in dogs in 1979 in Botswana [256], while the first anatomo-pathological description dates to 2003. At necropsy, gross pathology atrial epicarditis, hepatopathy, pulmonary edema, and spleen acute infarcts were observed, while no pathological remarks were reported in the CNS. At histopathology, the most prominent features were encephalitis and malacia of the basal ganglia. Myocarditis was also detected [257].

Natural WNV infection was also reported in the wolf. At necropsy gross pathology was inappreciable, while at histopathology WNV-related lesions were observed. Perivascular lymphocyte cuffing, foci of lymphocyte aggregates dispersed in the white and gray matters, and microglial nodules were recorded. Other microscopic lesions were myocarditis and very small foci of necrosis and lymphocyte infiltrates in the zona glomerularis of the adrenal gland cortex [257].

Cats can become infected through mosquito bites or by eating infected small mammals (demonstrated experimentally in mice) or birds [257,258].

(b) Experimental infection

Experimental WNV infection in dogs and cats was performed, and viremia was detected. At necropsy, no gross pathology was recorded in the dog, while WNV-infection-unrelated gross lesions were observed in cats such as hydrocephalus and spleen nodular fibrosis [257]. No histopathology was reported.

No evidence of intraspecies WNV transmission in companion animals or interspecies transmission between companion animal and humans [257].

#### 14.4.2. Carnivores (Wildlife)

##### Natural Exposure and Natural Infection

In bears, the majority of WNV exposure data regards serological investigations, except for a pathological study performed in one captive Polar bear (*Ursus maritimus)*.

The first data on WNV exposure in the bear came from a study by Madić and co-workers in Croatia in 1993 [259]. The serological survey was planned in 22, 13 wild and 9 captive, European brown bears (*Ursus arctos*). Serological positivity was detected in 4 out of 15 bears [259].

In 2002 in New Jersey, in 6 out of 51 free-ranging healthy black bears (*Ursus americanus*), a serological investigation for WNV allowed for the detection of NAs [260], while in Wisconsin, in a two-year period (February 2003–March 2005), the serological positivity was detected in 13 out of 71 free-rooming healthy black bears [261]. Out of 24 free-ranging European brown bears from Slovakia, 1 bear was seropositive for WNV [262].

A WNV serological and virological investigation, in free-ranging healthy European brown bears, in the Kars province in Turkey, showed seronegativity, and WNV nucleic acid was not found in any biological sample.

A Polar bear at the Toronto Zoo presented clinical signs of acute non-ambulatory paraparesis [263]. No data regarding gross pathology were described, while at histopathology, a mild-to-moderate non-suppurative meningoencephalomyelitis was observed. CNS at immunohistochemistry investigation returned a negative response, but in a retrospective study performed on spleen, a weak and rare WNV immunopositivity, probably in macrophages, was detected [263].

#### 14.4.3. Mesocarnivores

WNV natural infection was recorded in many Families of mesocarnivores, such as Canidae (coyotes, foxes), Viverridae (civets), Mustelidae (martens), Procyonidae (raccoon), and Mephitidae (skunks).

##### Canidae (Coyote—Fox)

In five Nebraska ecoregions, free-ranging for coyotes, a WNV serological survey was performed, collecting 67 sera (*Canis latrans*), during a 3-month campaign (November 2002–January 2003). In 48% of sera, antibodies for WNV were titrated [110].

In another study performed in South Dakota, where coyotes were used as sentinel animals for assessing the decline of the number of Black foot ferrets (*Mustela nigripes*), 71% was seropositive for WNV. The authors hypothesized that wide exposure to WNV in coyotes could be likely due to black-footed ferrets. It was emphasized that the sero-epidemiological condition detected in the coyote population needs to be investigated to understand a possible cause–effect relationship for the decline of the black-footed ferrets [264].

In both studies conducted in Nebraska and South Dakota, no data were available concerning gross pathology or histopathology.

WNV infection in Red fox (*Vulpes vulpes*) was performed in central Spain (Doñana National Park), and 21 out of 103 foxes were serologically positive for WNV antigens [248]. No data were available concerning gross pathology or histopathology.

##### Viverridae

In 1968, during a serological and virological study on wildlife in Ethiopia, a seropositivity for WNV antigens was detected in a Civet (*Civettictis* spp.) caught in Manera [248]. No data were available concerning gross pathology or histopathology.

##### Mustelidae

In a sero-surveillance investigation performed in central Spain (Doñana National Park), a stone marten (*Martes foina*) tested positive [248]. No data were available concerning gross pathology or histopathology.

In a two-year-old male Sea otter (*Enhydra lutris nereis*) showing inappetence, a moderate leukopenia, monocytosis, and an increase in CK were detected. WNV serology was strongly positive.

At necropsy, for gross pathology, the most prominent features were lymphoadenomegaly and congestion of meningeal bloody vessels. At histopathology, a severe diffuse meningoencephalomyelitis was recognized, associated with lymphoplasmacytic perivascular cuffing neuronal necrosis and neuro-satellitosis [253].

#### 14.4.4. Procyonidae

(a) Natural Exposure

Antibodies with seroprevalence of 34% were detected in raccoons (*Procyon lotor*) [111].

(b) Experimental Infection

In experimental infection in raccoons, viremia was registered, as well as protracted fecal shedding, but the virus was not detected in tissues, and histopathology was not referable to WNV infection [48].

#### 14.4.5. Mephitidae

In 2001, WNV infection was confirmed in an Eastern striped skunk (*Mephitis mephitis*) from Fairfield County, Connecticut. No data were available concerning gross pathology and histopathology.

#### 14.4.6. Order: Lagomorpha

##### Rabbit

Spontaneous infection in rabbits was not cited so far, but two species of rabbits, New Zealand White (*Oryctolagus cuniculus*) and North American cottontail (*Sylvilagus* spp.), were experimentally infected with WNV. An experimental investigation showed that rabbits were immune-resistant to WNV, confirmed by low viremia. The neuroinvasion was minimal to absent, as well as mild to moderate. Nervous lesions ranged from mild to moderate mononuclear leukocytic CNS infiltration, perivascular lymphocyte cuffing, and gliosis. Neuronal degeneration and neuronophagia were infrequent. Extra-neural WNV replication takes place in lymph nodes, in which lymphocyte hyperplasia was recorded in germinal lymphoid centers [265].

#### 14.4.7. Order: Marsupialia

The first WNV detection in a marsupial was referred by Bosco-Lauth and co-workers in 2014 [266] from the blood of a Virginia opossum (*Didelphis virginiana*), in Missouri. A WNV genetic molecular array framed the virus to lineage 1a WNV02 strains [266].

Only in 2017, Lamglait and Lair [267] provided detailed gross pathology and histopathology descriptions in a Virginia opossum in a zoological setting (Montreal region—Quebec), which was affected by pulmonary adenocarcinoma.

The most relevant clinical sign reported in the case history was a mild pulmonary whistle.

At necropsy, multifocal solid-white nodules were recognized in the lungs diagnosed at histopathology as pulmonary lepidic adenocarcinoma. Other gross lesions recognized in the myocardium as multifocal tan discoloration and mild splenomegaly. Histologically, CNS (telencephalon, and cerebellum) was characterized by a diffuse meningoencephalitis, lymphoplasmacytic perivascular cuffing and neuropil vacuolization. At histopathology, the myocardium was featured by severe multifocal necrotizing myocarditis with large areas of lymphoplasmacytic infiltration. A necrotic myositis was also recorded in *M. triceps brachii* and *M. quadriceps femoris* [267,268].

A genetic molecular investigation for WNV reverse transcription RNA on a pool of tissues—samples collected from the brain, myocardium, and spleen—was positive [267].

#### 14.4.8. Zoo Animals

Many serological WNV investigations were performed in zoo animals in the USA, Mexico, and Europe (Spain, Slovenia, France):

(a) USA

The first survey started at Wildlife Conservation Society/Bronx Zoo (WCS) in August 1999 (1999–2000) in several avian species and mammals in a zoological setting. In mammals, WNV antibodies were detected only outdoors, equal to 5.9% (raw data: 6 out 101), in the following species: two Indian elephants (*Elephas maximus indicus*), one greater Indian rhinoceros (*Rhinoceros unicornis*), one Babirusa (*Babyrousa babyrussa*), one Lesser panda (*Ailurus fulgens fulgens*), and one Snow leopard (*Panthera uncia*). No WNV antibodies were detected in the housed donkey (*Equus asinus asinus*) and Przewlaski’s horse (*Equus ferus przewalskii*) [269]. No data were available concerning gross pathology or histopathology.

(b) Mexico

A serosurveillance campaign (2003–2004) for evidence of WNV was performed collecting blood from 415 vertebrates (257 birds, 52 mammals, and 106 reptiles) belonging to 61 species from the Merida Zoo, Yucatan, and 7 farmed crocodiles in Ciudad del Carmen, Campeche. Seven birds and two mammals (jaguar and coyote) were seropositive for WNV by PRNT, and 6 out of 7 (86%) crocodiles were PRNT-confirmed for WNV infection. No evidence of clinical signs in any semiaquatic reptiles tested was reported [270]. No data were available concerning gross pathology or histopathology.

Puerto and co-workers in 2008 [271] reported data regarding WNV in zoo animals and employees from Tabasco and Yucatan, Mexico. One serosurvey regarded 270 animals under zoological setting at the Tabasco Zoo, and the other involved 151 animals at the Yucatan Zoo. Sera of sixty employees were also tested. Sera collected in animals from the Tabasco Zoo showed a WNV positivity equal to 26.11% in birds, 38.09% in mammals, and 71.42% in reptiles. In the Yucatan Zoo only, avian species were positive for WNV antibodies, equal to 7.6% of the birds tested, while mammals and reptiles were WNV seronegative. Sera tested in employees were negative for RT-PCR and blocking ELISA [271]. The authors highlighted that the contact of employees-zoo animals was limited to daylight hours. No data were available concerning clinical sings, gross pathology, or histopathology.

(c) Spain

In Spain, between 2002 and 2019, a serosurvey was performed to check the presence of Orthoflavivirus in ten zoos. Blood samples were collected in 570 zoo mammals belonging to 120 species. WNV neutralization test was positive in three African elephants (*Loxodonta africana*), one Barbary macaque (*Macaca sylvanus*), one Giant panda (*Aliuropara melanoleuca*), and five Thomson’s gazelle (*Eduorcas thompsonii*) [272]. No data were available concerning clinical signs, gross pathology, or histopathology.

(d) Slovenia

A WNV serosurvey was performed between 2002 and 2018 on sera of 501 animals under a zoological setting in Ljubljana Zoo. Blood was collected from 261 animals of 84 animal species, including birds, rodents, lagomorphs, carnivores, ungulates, reptiles, equids, and primates. WNV tested positive at virus neutralization test in avian species, but no mammals presented WNV-NAs [103]. No data were available concerning clinical sings, gross pathology, or histopathology.

(e) France

A WNV serosurvey was performed in two zoos in the south of France (Montpellier and Sigean) between 2002 and 2019 on the sera of 411 animals. Blood was collected from 70 species. In mammals, WNV antibodies were detected in one Dama gazelle (*Nanger dama*) [102]. No data were available concerning clinical signs, gross pathology, or histopathology.

#### 14.4.9. Order: Rodentia

##### Squirrel

(a) Natural Infection

Fox squirrel (*Scirius niger*) showed motor neurologic signs varying from involuntary movements to muscular paralysis. At necropsy, gross pathology was unapparent, while histopathology was relevant for CNS lymphoplasmacytic inflammation. Same histopathological features were also recognized in the heart, kidney, and liver [273].

(b) Natural Exposure

Natural exposure to WNV was detected in many world areas in squirrels belonging to the family Sciuridae: Fox squirrel (*Scirius niger*) [111,274,275,276,277,278,279], Red squirrel (*Tamiasciuris hunsonicus*) [278,280], Easter gray squirrel (*Sciuris carolensis*) [35,275,277,281], Western gray squirrel (*Sciuris griseus*) [276,279], and Eastern chipmunk (*Tamias striatus)* [35,277,279]. Antibodies to WNV were detected both in dead and alive healthy squirrels. No descriptions were available regarding gross pathology and histopathology investigations. In Eastern chipmunk, the seroprevalence was low in comparison with that recorded in other squirrel species.

(c) Experimental Infection

Many experimental WNV infections in Fox squirrel (*Tamasciuris hunsonicus*) were performed for investigating various aspects of WNV transmission [279,280]. Gross pathology was unapparent in 16 out of 17 necropsied squirrels. In one squirrel, at histopathology, non-suppurative encephalitis in the telencephalon and rhombencephalon was recorded with prominent features of multifocal gliosis and lymphocytic perivascular cuffing. Myocardial lesions were lymphocytic infiltration (2 squirrels) and fibrosis (4 squirrels). Interstitial lymphocytic nephritis was recorded in infected squirrels (3 out of 4) and uninfected controls (1 out of 4). Periportal mononuclear leukocytes in the liver was a common feature in infected and uninfected squirrels [280].

In the study by Tiawsirisup and co-workers [274], no data for gross pathology were given, while histopathology was non-representative for classical WNV infection, except in one Fox squirrel [273].

##### Rat and Mouse

(a) Natural Exposure

In *Rattus* spp. and *Mus* spp., natural exposure was detected in a serosurveillance investigation with antibody quantification, without any description concerning gross pathology or histopathology [279].

(b) Experimental Infection

Garcia-Tapia and co-workers [282] performed an experimental study in mice for describing the histopathological changes in the CNS over time during WNV infection: glial–microglia nodules, perivascular infiltration, neuronal necrosis, neuronophagia, and neuro-satellitosis.

Neuro-histopathology was recorded not before 6 dpi (glial–microglia nodules, perivascular infiltration, satellitosis), while neuronal necrosis and neurophagia became present three days later (9 dpi). Neurons were positive at immunohistochemistry at 6 dpi till 21 dpi (last day of the experimental period) [282].

Many rodents were exposed to WNV and produced antibodies, as reported by Root [279] in 2013, but no data were available regarding gross pathology or histopathology in the following animal species: Indian gerbil (*Tatera indica*), Bushy-tailed jird (*Sekeetamys calurus*), Wagner’s dipodil (*Dipodillus dasyurus*), Greater Egyptian gerbil (*Gerbillus pyramidum*), Guenther’s vole (*Microtus guentheri*), Bank vole (*Myodes glareolus*), Common vole (*Microtus arvalis*), Common hamsters (*Cricetus cricetus*), Common gundi (*Ctenodactylus gundi*), gerbil (Gerbillus spp.), woodchuck, or groundhog (*Marmota monax*).

## 15. Reptilia

Reptiles are receptive to WNV infection, showing viremia and developing an immune response. In laboratory settings, WNV infection was recorded in Green Iguanas (*Iguana iguana*), Eastern Garter Snakes (*Thamnophis sirtalis sirtalis*), Red-Ear Sliders (*Trachemys scripta elegans*), North American Bullfrogs (*Lithobates catesbeianus*) and Western Fence-Lizards (*Sceloporus occidentalis*) [283,284,285,286]. Eastern Garter Snakes (*Thamnophis sirtalis sirtalis*) tested positive for WNV RNA [231]. Crocodilians are the most receptive animals and cases in wildlife or in farmed animals have been recorded in Israel, USA, Mexico, and Zambia [107,255,270,287,288].

WNV outbreaks (2001–2003) caused large-scale mortalities in farmed American alligators (*Alligator mississippiensis*) in the southeastern USA [107,245,255]. The alligators were raised indoors and fed with a supplementation of horsemeat [243]. A genetic molecular investigation (RT-PCR) on the presence of WNV in horsemeat demonstrated positive meat samples during the WNV outbreak in 2002 [245].

Most receptive to WNV infection are the young alligators. Neuro-clinical signs are recorded, such as anorexia, muscular tremors, swimming on their sides, spinning in the water, and opisthotonos [287]. At necropsy, gross pathology can be limited to stomatitis. Histologically, the most relevant features were observed in farmed animals but not in free-ranging alligators.

At histopathology, multi-organ necrosis and heterophilic inflammation were the prominent features [107,245].

Brain and spinal cord meningeal lymphoplasmacytic infiltrates, mixed with rare heterophilic cell infiltrates as well as perivascular cuffs were recognized. Comparable cellular infiltrates were also observed in pulmonary and renal interstitia [287]. Multifocal myocardial foci of degeneration and lymphoplasmacytic and heterophilic infiltrates were described. Diverse inflammatory cells were detected in the lamina propria of the intestine [287]. Skin lesions, called “pix” were recorded in alligators and crocodiles. The lesions, 1–2 mm in size, appear translucent and more defined after skin tanning. At histological examination, a lymphohistiocytic population was recognized [12,289,290]. Gross pathology showed multifocal translucent lesions, 1–2 mm in size, which were more prominent after tanning [12,290].

## 16. Aves

Birds are the most susceptible animals to WNV infection. Serological surveys performed in many epidemiological campaigns have pointed out that over 326 avian species are positive for WNV antigens in different world geographical areas [46,291]. A WNV post-mortem monitoring performed in Central Italy counted sixteen Orders and twenty-eight Families [291].

Probably, the list of seropositive WNV-infected birds is destined to grow further. Raptors and Corvids, especially crows, jays, and ravens, are very susceptible to WNV infection. WNV was isolated in the oral cavities from Corvid carcasses experimentally infected [255].

In poultry and in turkeys, WNV infection is infrequent [46,292,293].

The highest bird receptivity to WNV infection occurs in a high percentage of cases, and the infection often progresses to a severe form, with evidence of neuro-muscular clinical signs and associated with histological lesions [46]. Often, in birds with high viraemia and rapid clinical/neuro-clinical evolution to death, the histological lesions may be inapparent under the light microscope due to the short time elapsed between infection and the bird death [113,158]. Moreover, the severity of lesions is related to host factors and intrinsic viral factor variability [158]. The clinical signs related to WNV infection are mainly neuro-clinical with evidence of neuro-muscular symptoms. At gross pathology during necropsy, tissue damages were not indicative [46,158,291] or unspecific, such as mild hepatomegaly or multiorgan congestion [291]. CNS lesions were described as lymphoplasmacytic, and histiocytic meningoencephalitis, gliosis, glial nodules, and perivascular cuffing were recognized in the telencephalon, mesencephalon, and rhombencephalon [46,158,291]. Neuronal necrosis and neurodegeneration were recorded in raptors [294] and owls [295].

The gastrointestinal tract, kidney, spleen, liver, pancreas, lung, adrenal glands, thyroid, thymus, bursa, bone marrow, and skeletal muscle were found also damaged [46,158]. Palmieri and co-workers [46], using an immunohistochemical approach, immunolocalized WNV antigen in the heart, lung, kidneys, proventricul, intestine, pancreas, liver, spleen, ovaries and oviducts, adrenal glands, testis, brain, skin, skeletal muscles, crop or esophagus, and thymus.

Raptor and owl eyes were investigated accurately due to the vital importance of eyes in these bird species. Lymphoplasmacytic and histiocytic infiltrates and retinal lesions were recorded in spontaneous WNV infection in hawks; disarray of the retinal pigmented epithelial cell layer and retinal cell necrosis and mineralization were described in naturally WNV-infected hawks [296].

Perivascular/vascular lesions were sporadically observed [46]. As aforementioned, poultry and turkey WNV infection is infrequent, but young chicks are more susceptible to infection [46,292,293]. The resistance to WNV infection in adult chicks is probably related to the age and immunocompetence. In case of WNV infection in adult chicks, the lesions are recognizable in the heart (myocardial necrosis), kidney (nephritis), and lung (pneumonitis), while brain infection (encephalitis) is not common [292].

## 17. Economic Impact and Climate Change

In veterinary medicine, the study on the economic impact related to WNV began after the outbreak dated to 1999 in the Bronx Zoo, approximately sixty years after its discovery in Uganda in 1937. The first paper (Info sheet) dated to April 2003 prepared by the Animal and Plant Health Inspection Service (APHIS) on the Colorado and Nebraska equine industry [297], which was followed over the years by comparable studies conducted in Louisiana [298], North Dakota [299], California [300], and overall in the USA [7,301]. The themes were related to costs of death, loss of use, and treatment (therapies and vaccine prophylaxis). Other mixed studies on humans and horses were performed in Europe: Greece [302], Belgium [303], Italy [304], and in Southern and Southeastern Europe [71], pointing out the amount of the direct healthcare costs in both species. A recent study was conducted in Ontario (Canada) based on laboratory and health administrative data [305]. Research on economic impact performed in low- and middle-income countries related to mosquito-borne diseases was directed to analyze the effect on their Gross Domestic Product (GDP) [306].

To better make sense of the economic impact in the decades to come, it is useful to set up the research by making a parallel between the change in climate conditions and the potential spread of the areas for insects of the Genus Culicoides [7,33,71,307,308]. In conditions of territorial control of the spread of WNV infection, the major expenses are to be attributed to the control of the spread of populations of avian species (primary host), to the entomological studies (hematophagous vectors), and to the monitoring of cases in equine species (secondary host) [29,71].

The most significant interest and economic impact arose after the recognition in the 1990s of the new neuroinvasive WNV strain (lineage 1) in Romania and then in other European countries, as well as Russia and Israel, despite WNV infection as classical clinical forms (i.e., subclinical or febrile) had occurred in the African continent, Middle Eastern countries, and in Europe for about sixty years [215].

## 18. WNV Surveillance on the Territory

The WNV surveillance approach is based on monitoring the territory to evaluate the Eco-Health risk for protection of One-Health.

In Italy, according to the National Plan for the Prevention, Surveillance, and Control of Arboviroses (“Piano Nazionale Arbovirosi” [PNA]) 2020–2025, surveillance can be divided into passive and active. Passive surveillance is compulsory throughout the country and includes (a) surveillance of cases of nervous symptoms in equidae; (b) surveillance of wild birds found dead; and (c) surveillance of cases of neuroinvasive disease and/or recent human infections.

Active surveillance is compulsory in areas where the risk of circulation of WNV is medium or high and includes (a) surveillance of resident birds belonging to target species; (b) entomological surveillance; and (c) serological surveillance in rural or free-range poultry flocks.

Surveillance of hematophagous insects transmitting WNV is based on insect-trapping systems.

The first system is capable of capturing insects belonging to the genus *Culex* spp. using insect traps such as the Centers for Disease Control light traps (CDC-LT) or modified CDC trap types, which are CO_2_-activated and particularly suitable for capturing adult specimens belonging to different crepuscular and nocturnal Culicidae species. Another insect-trapping system, called Gravid, is used to capture pregnant female mosquitoes that need to lay their eggs and approach the water. This makes it possible to capture adult females that have completed and digested at least one blood meal and can transmit WNV through salivary gland secretions [309].

Samples collected from animals (e.g., body fluids or tissues) or from captured hematophagous insects are investigated using immunological and molecular methods (e.g., ELISA, RT-PCR) at accredited national diagnostic centers. In Italy, the diagnostic procedures are based on a double step system. The unknown samples are first tested in one of the territorial laboratories of the Department of Health, Istituto Zooprofilattico Sperimentale; then, the positive case must be confirmed by the National Reference Centre for the study and diagnosis of exotic animal diseases (“Centro di Referenza Nazionale per lo studio e l’accertamento delle malattie esotiche degli animali”, CESME) [309].

## 19. Foundations of the European WND Legislation

WND is regulated by various European directives that outline surveillance protocols, control measures, and response actions for suspected or confirmed cases.

The main regulatory framework is Regulation (EU) 2016/429 of the European Parliament and of the Council of 9 March 2016 on transmissible animal diseases, amending and repealing certain acts in the area of animal health, known as the “Animal Health Law”, which establishes a cohesive legal framework for animal health throughout the European Union. This regulation supports the One-Health strategy and consolidates health regulations through a streamlined and flexible approach.

Another essential reference is Implementing Regulation (EU) 2018/1882 (Commission Implementing Regulation (EU) 2018/1882 of 3 December 2018 on the application of certain disease prevention and control rules to categories of listed diseases and establishing a list of species and groups of species posing a considerable risk for the spread of those listed diseases), which categorizes animal diseases into five groups (A, B, C, D, E) based on the principles outlined in Article 7 (Assessment parameters for the listing of diseases) of Regulation (EU) 2016/429. WND falls into Category E, necessitating targeted surveillance across the European Union.

While primarily focused on Category B and C diseases, Delegated Regulation (EU) 2020/689 (Commission Delegated Regulation (EU) 2020/689 of 17 December 2019 supplementing Regulation (EU) 2016/429 of the European Parliament and of the Council as regards rules for surveillance, eradication programs, and disease-free status for certain listed and emerging diseases) also pertains to WND, delineating the structure of surveillance (Article 3—Design of surveillance), the target animal population and diagnostic tools (Article 4—Targeted animal population), as well as definitions for suspected and confirmed cases (Article 9—Case definitions).

As WND can be transmitted through donated blood, tissues, cells, and organs, it is essential to ensure the safety of Substances of Human Origin (SoHO); the European regulatory framework includes the following:

- Directive 2002/98/EC (Blood Directive) (DIRECTIVE 2002/98/EC OF THE EUROPEAN PARLIAMENT AND OF THE COUNCIL of 27 January 2003, setting standards of quality and safety for the collection, testing, processing, storage, and distribution of human blood and blood components and amending Directive 2001/83/EC);
- Directive 2004/23/EC (Tissues and Cells Directive) (DIRECTIVE 2004/23/EC OF THE EUROPEAN PARLIAMENT AND OF THE COUNCIL of 31 March 2004 on setting standards of quality and safety for the donation, procurement, testing, processing, preservation, storage, and distribution of human tissues and cells);
- Directive 2010/45/EU (Organs Directive) (DIRECTIVE 2010/45/EU OF THE EUROPEAN PARLIAMENT AND OF THE COUNCIL of 7 July 2010 on standards of quality and safety of human organs intended for transplantation).

After evaluating these directives, the EC intends to adopt a new quality and safety standards regulation for SoHO, which will replace Directives 2002/98/EC and 2004/23/EC.

Directive 2004/33/EC (Commission Directive 2004/33/EC of 22 March 2004 implementing Directive 2002/98/EC of the European Parliament and of the Council as regards certain technical requirements for blood and blood components) establishes a 28-day deferral period for donors returning from areas with active virus transmission to reduce the risk of virus transmission through donations. Alternatively, WNV Individual Donor Nucleic Acid testing (ID-NAT) can be performed to exclude the presence of the virus.

## 20. Definition of Case and Suspicion of Disease

A suspected case can be identified by the presence of at least one of the following criteria:

- clinical symptoms;
- anatomo-pathological lesions;
- positive laboratory test results;
- epidemiological correlation.

A confirmed case needs the following:

- isolation of the pathogen;
- the presence of symptoms or lesions in conjunction with a positive test;
- a positive test result accompanied by epidemiological correlation.

The surveillance of WND in humans is governed by Implementing Decision (EU) 2018/945 (Commission Implementing Decision (EU) 2018/945 of 22 June 2018 on the communicable diseases and related special health issues to be covered by epidemiological surveillance as well as relevant case definitions), which includes the disease on the list of communicable diseases to be monitored at the European level. Additionally, Regulation (EU) 2022/2371 (Regulation (EU) 2022/2371 of the European Parliament and of the Council of 23 November 2022 on serious cross-border threats to health and repealing Decision nr. 1082/2013/EU) establishes specific measures for managing cross-border health threats, including WND, requiring Member States to adopt appropriate surveillance and response strategies.

## 21. International Notification Obligations

WNF is a notifiable disease in equids and birds. According to the Terrestrial Code of the WOAH, the disease must be reported to WOAH through the WAHIS.

All EU notifications of WNV outbreaks in animals are initially recorded in the ADIS of the EC. This notification process indirectly supports the WAHIS, thereby disseminating disease information at the WOAH level.

Data from passive and active surveillance activities, including outbreaks, are collected by the European Food Safety Authority (EFSA) and analyzed alongside data from the ECDC for publication in the European Union One Health Zoonoses Report.

The complex European and national regulatory framework ensures a comprehensive approach to managing WND through the surveillance, control measures, and the safety of substances of human origin. Collaboration between veterinary and human health authorities, data collection, and epidemiological analysis serves as a fundamental pillar for preventing and responding to this zoonosis.

## 22. History and Legislation of WND in Italy: The Italian One-Health Prevention Model

The history of WNV in Italy began in 1998, when the virus was isolated for the first time in the provinces of Florence, Pisa, Lucca, and Pistoia (“Padule di Fucecchio”, the largest internal marsh in Italy, about 2000 hectares wide) [310]. The most accredited hypothesis on its onset suggested that migratory birds coming from sub-Saharan Africa, during stops in Italian wetlands, transmitted the virus to resident birds through ornithophilous mosquitoes.

This first appearance led Italy to establish, as early as 2002, a veterinary surveillance plan with the aim of monitoring the possible entry and circulation of the virus in the territory. This plan, in force until 2008, was subsequently updated in 2014 to respond to epidemiological changes and the knowledge gradually acquired. Traces of WNV were searched in endemic areas, near the outbreaks observed in previous years and in areas at high risk due to the presence of migratory birds and vectors. In 2016, with the adoption of the integrated national plan, collaboration between the medical and veterinary sectors was formalized, thus improving the ability to identify the circulation of the virus early and estimate the health risks for humans.

The turning point came in 2018, the year in which Italy recorded an unprecedented wave of human cases of WND: 577 infections and 42 deaths, which represented about half of the European cases [310]. This crisis has highlighted the urgency of a coordinated and structured approach, thus leading, in 2020, with act nr. 1, on 15 January 2020, thanks to the agreement between the Government, Regions and the autonomous Provinces of Trento and Bolzano, to the adoption of the Italian PNA 2020–2025 [311].

This strategic document, divided into 8 chapters and 18 annexes, aims to consolidate the One-Health model to address a problem that affects not only public health but also animal and environmental health according to the concept developed by the New Delhi International Ministerial Conference in 2007, which, following global health emergencies, highlighted the urgency of integrated strategies to address pandemic threats.

Drawing inspiration from the WHO Resolution of the World Health Assembly (WHA 70.16), which invited States to develop plans for vector control, and from the EU directives, which updates the list of diseases to be incorporated into the community epidemiological surveillance network, including arboviroses and other vector-borne diseases, the Italian PNA 2020–2025 plan represents a complex and multidisciplinary response [311].

Recently, in 2023 (ministerial circular nr. 31185 of 12 November 2023 signed by the General Director of the former General Directorate of Animal Health and Veterinary Medicines, “Direzione Generale della Salute Animale e dei Farmaci Veterinari”, i.e., DGSAF), the Ministry of Health, also following the entry into force of EU regulation 2016/429 and Legislative Decree 136/2022, has adapted and further refined the operational strategies by issuing guidelines to strengthen territorial veterinary activities, starting from mandatory regional programming, confirming the Italian commitment to combating arboviroses in assiduous collaboration with the category of human doctors. This last aspect is the reference of the joint ministerial circular note between the former General Directorate of Animal Health and Veterinary Medicines and the former Prevention Directorate released on 7 June 2023 and protocol nr. 0017581 with the subject “Arbovirus surveillance and response, general indications”.

The next revision of the plan will be the simplified but impactful evolution in the fight against arboviroses, highlighting how the One-Health model today represents an essential necessity for national and global health security. The involvement of everyone, from citizens to stockholders to private and public professionals, will be fundamental and an opportunity for everyone to implement prevention.

## 23. Conclusions

The One-Health and Eco-Health emergencies have led the international health community to build a flexible and effective system to control and contain the territorial circulation of WNV, especially in endemic countries. Surveillance strategies are geared towards monitoring the serological presence of WNV in birds and insect vectors, using laboratory methods also to draw a dynamic epidemiological map of endemic areas.

Under the conditions of WNV control and containment, the greatest economic impact is related to serological monitoring and laboratory costs, while during outbreaks, the costs of treatment are related to hospitalization of humans and vaccination for equines. In addition, the risk for travelers and tourists is unclear and economically unquantifiable, especially in counties where climate, and human and animal lives are highly interconnected, and may also limit the smooth running of local and international equestrian events (e.g., Olympic Games; Pushkar Fair, Pushkar (Ajmer district), Rajasthan, India [Ferrari L., personal observations, Pushkar, November 2024]). It is useful to remember that for veterinary pathologists, there is a risk of infection while performing the necropsy of WNV-infected horses. The epidemiological and evolutionary analysis of WNV is the main approach to motivate sanitary and ecological actions that are the direct and logical consequence to contain the risks associated with WNV and WND.

## Data Availability

Not applicable.
